# 2-Hydroxysorangiadenosine: Structure and Biosynthesis of a Myxobacterial Sesquiterpene–Nucleoside

**DOI:** 10.3390/molecules25112676

**Published:** 2020-06-09

**Authors:** Dorothy A. Okoth, Joachim J. Hug, Ronald Garcia, Cathrin Spröer, Jörg Overmann, Rolf Müller

**Affiliations:** 1 Department Microbial Natural Products, Helmholtz-Institute for Pharmaceutical Research Saarland (HIPS), Helmholtz Centre for Infection Research (HZI) and Department of Pharmacy, Saarland University, Campus E8 1, 66123 Saarbrücken, Germany; Dorothy.Okoth@helmholtz-hips.de (D.A.O.); Joachim.Hug@helmholtz-hips.de (J.J.H.); Ronald.Garcia@helmholtz-hips.de (R.G.); 2 German Center for Infection Research (DZIF), Partner Site Hannover-Braunschweig, 38124 Braunschweig, Germany; joerg.overmann@dsmz.de; 3 Leibniz Institute DSMZ-German Collection of Microorganisms and Cell Cultures, 38124 Braunschweig, Germany; Cathrin.Sproeer@dsmz.de

**Keywords:** myxobacteria, terpene–nucleoside, biosynthesis, antibiotics, sorangiadenosine, 2-hydroxysorangiadenosine, secondary metabolites, genome-mining, antibiotics, natural products discovery

## Abstract

Myxobacteria represent an under-investigated source for biologically active natural products featuring intriguing structural moieties with potential applications, e.g., in the pharmaceutical industry. Sorangiadenosine and the here-discovered 2-hydroxysorangiadenosine are myxobacterial sesquiterpene–nucleosides with an unusual structural moiety, a bicyclic eudesmane-type sesquiterpene. As the biosynthesis of these rare terpene–nucleoside hybrid natural products remains elusive, we investigated secondary metabolomes and genomes of several 2-hydroxysorangiadenosine-producing myxobacteria. We report the isolation and full structure elucidation of 2-hydroxysorangiadenosine and its cytotoxic and antibiotic activities and propose a biosynthetic pathway in the myxobacterium *Vitiosangium cumulatum* MCy10943^T^.

## 1. Introduction

Myxobacteria are remarkable Gram-negative bacteria exhibiting several unusual characteristics, such as coordinated movement by gliding and creeping on surfaces in a swarm-like pattern [1]. Unlike other prokaryotes, they show unique cooperative “social behavior”, based on complex chemical communication systems [2]. A wide variety of modes of action have been documented for myxobacterial secondary metabolites with diverse bioactivities [3], such as the antifungal soraphen A [4], the antibacterial cystobactamid 919-2 [5], the antiplasmodial chlorotonil [6], the antiviral aetheramides [7] and the cytotoxic pretubulysin [8,9]. Most of these compounds derive biosynthetically from giant biosynthetic enzyme complexes such as modular polyketide synthases (PKSs), non-ribosomal peptide synthetases (NRPSs) and hybrids thereof, while natural products from other biosynthetic machineries have been less frequently isolated.

Terpene natural products in myxobacteria have primarily been investigated by analysis of the volatiles emitted from cell cultures of *Myxococcus xanthus* sp. [10] and *Stigmatella aurantiaca* sp. [11] through a closed-loop stripping apparatus and subsequent gas chromatography–mass spectrometry (GC-MS) analysis. These investigations revealed not only the biosynthesis of several myxobacterial volatiles through stable isotope-labeled precursor feeding but also permitted further insights into the formation of the well-known terpenes geosmin [12] and 2-methylisoborneol [13,14]. The biosynthetic characterization of (+)-eremophilene biosynthesis [15] and the function of terpene synthase Soce6369 (10-*epi*-cubenol synthase) [16] in *Sorangium cellulosum* Soce56 provided additional in-depth knowledge of terpene biosynthesis in myxobacteria.

The sesquiterpene adenoside sorangiadenosine (**1**) has been isolated from the crude organic extract of *Sorangium cellulosum* KM1003 [17]. Evaluation of its biological function indicated moderate antibacterial activity against the test strains *Staphylococcus aureus* ATCC 6538p, *Bacillus subtilis* ATCC 6633, *Micrococcus luteus* IFC 12708, *Proteus vulgaris* ATCC 3851, *Salmonella typhimurium* ATCC 14028, and *Escherichia coli* ATCC 25922 [17]. Our current interest was focused on deciphering the biosynthetic logic of this terpene–nucleoside hybrid. Terpene–nucleoside hybrids are a rarely described class of natural products, reflected by the fact that **1** is the only known terpene–nucleoside hybrid from a myxobacterium.

**Figure 1:**
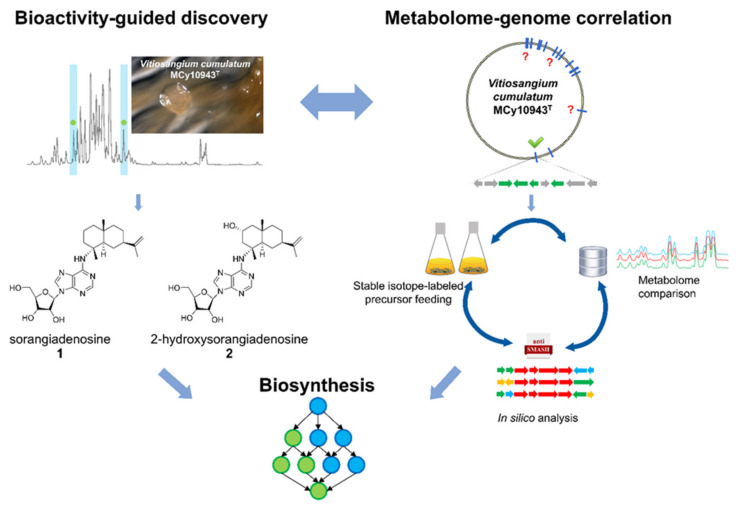
Bioactivity-guided metabolome screening of *Vitiosangium cumulatum* MCy10943^T^ led to the re-discovery of sorangiadenosine (**1**) and the discovery of 2-hydroxysorangiadenosine (**2**). The molecular weight, sum formula and MS^2^ fragmentation pattern of the unknown molecules enabled identification and isolation of **1** and **2**. The corresponding biosynthetic gene cluster was identified via in-silico investigation of the MCy10943^T^ genome sequence. Stable isotope-labeled precursor feeding experiments and comparative metabolome investigation of genome-sequenced myxobacterial strains corroborated this analysis. Finally, the outcome of this study led to a proposed biosynthetic route responsible for the biosynthesis of these unique sesquiterpene–adenosine hybrids.

We report here the isolation and the cytotoxic and antibacterial activities together with the full structure elucidation of the new sorangiadenosine derivative 2-hydroxysorangiadenosine (**2**) from the myxobacterial producer *Vitiosangium cumulatum* MCy10943^T^ [18], which belongs to a different myxobacterial family than the previously described producer of **1**. In addition, genome sequencing of the producer strain *V. cumulatum* MCy10943^T^ and comparative metabolome analysis of several additional myxobacterial strains led to the identification of a putative biosynthetic gene cluster (BGC) involved in the formation of **1** and **2** (Figure 1).

## 2. Results

### 2.1. Discovery of 2-Hydroxysorangiadenosine and Sorangiadenosine

**Figure 2:**
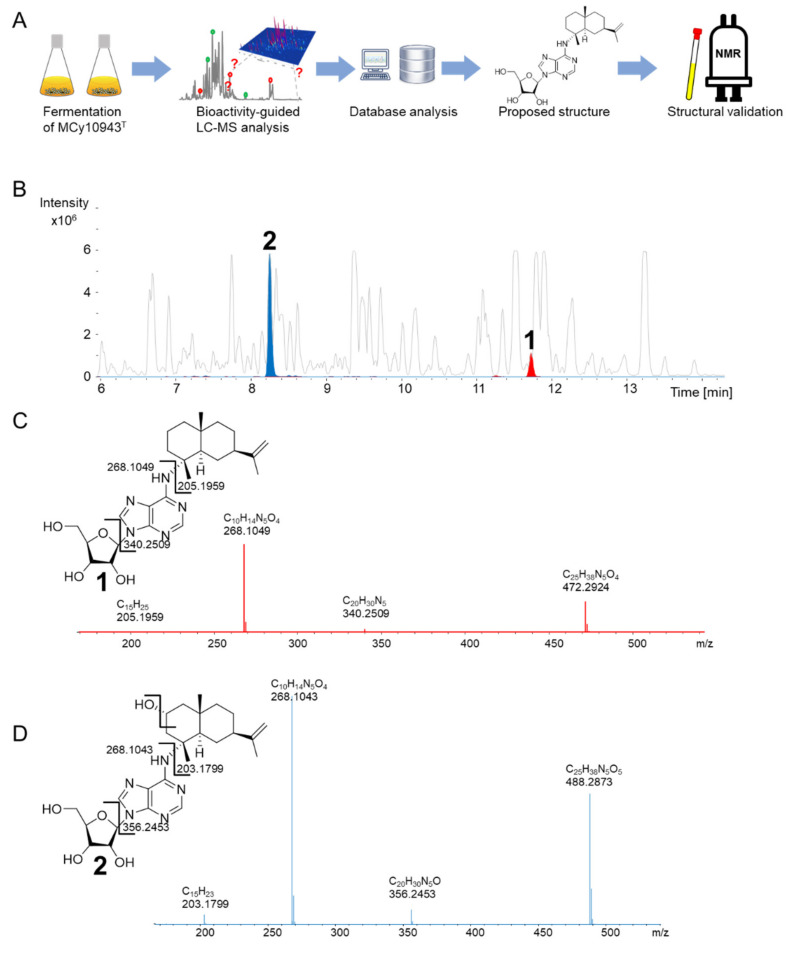
(**A**) Workflow of secondary metabolite screening in MCy10943^T^, resulting in the independent re-isolation of sorangiadenosine (**1**) and the discovery of 2-hydroxysorangiadenosine (**2**). (**B**) High-performance liquid chromatography–mass spectrometry base peak chromatogram (HPLC-MS BPC) (grey) and extracted ion chromatogram (EIC) of **1** ([M + H]^+^ 472.2923 *m*/*z*, red) and **2** ([M + H]^+^ 488.2873 *m*/*z*, blue) from MCy10943^T^ crude extracts. Fragmentation of sorangiadenosine (**C**) and 2-hydroxysorangiadenosine (**D**) in MS^2^ experiments.

**Figure 3:**
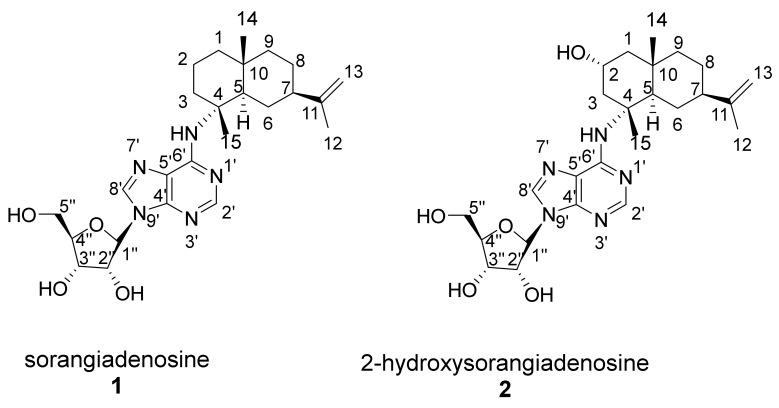
Chemical structure and numbering of sorangiadenosine (**1**) and 2-hydroxysorangiadenosine (**2**).

In the course of our bioactivity-guided secondary metabolite screening of the recently isolated strain *V. cumulatum* MCy10943^T^ [18] (Figure 2A), high-performance liquid chromatography (HPLC)-fractions of the crude extract active against *Bacillus subtilis* revealed two target masses. One secondary metabolite features an ion peak in the liquid chromatography–mass spectrometry (LC-MS) chromatogram at mass-to-charge ratio (*m*/*z*) 472.2924 [M + H]^+^, supporting the deduced molecular formula C_25_H_38_N_5_O_4_ at the retention time of 11.72 min, while the second target mass features an ion peak at *m*/*z* 488.2873 [M + H]^+^, supporting the deduced molecular formula C_25_H_38_N_5_O_5_ at the retention time of 8.24 min (Figure 2B). A search in several chemical structure databases revealed that the target mass of 472.2924 [M + H]^+^ might correspond to the previously isolated natural product **1**, whereas the second natural product **2** could be a derivative thereof, according to the observed tandem mass spectrometry (MS^2^) fragmentation pattern of both compounds. The MS^2^ fragment of **1** was characterized by *m*/*z* 340.2509 (C_20_H_30_N_5_^+^), 268.1047 (C_10_H_14_N_5_O_4_^+^) and 205.1959 (C_15_H_25_^+^), consistent with [M + H + sugar]^+^, adenosine and sesquiterpene [M + H]^+^ fragment ions (Figure 2C). Similarly, the MS^2^ fragment of compound **2** was characterized by *m*/*z* 356.2453 [M + H]^+^ (C_20_H_30_N_5_O^+^), *m*/*z* 268.1043 (C_10_H_14_N_5_O_4_^+^) and *m*/*z* 203.1799 (C_15_H_23_^+^) consistent with [M + H + sugar]^+^, [adenosine + H]^+^, and [M + H + adenosine + OH]^+^ ion peaks (Figure 2D). This suggested that both compounds were made of a sesquiterpene, adenine and a sugar moiety, with compound **2** having an additional oxygen atom (Figure 2C,D). Since *S. cellulosum* KM1003, the original myxobacterial producer of **1**, was not deposited in a publicly accessible collection, and a reference substance was not commercially available, the identity of the detected compound was validated via isolation and structure elucidation of the identified compounds. The recorded proton (^1^H) nuclear magnetic resonance (NMR) and carbon-13 (^13^C) NMR of both purified compounds proved the identity of these compounds as **1** and the new derivative **2** (Figure 3).

The ^1^H NMR and ^13^C NMR data of **1** was consistent with the observed correlations of the previously isolated sorangiadenosine from a *S. cellulosum* KM1003 [17]. The ^1^H and ^13^C-NMR data of **2** resembled that of **1**. The heteronuclear single quantum coherence/correlation (HSQC) and ^13^C-NMR of **1** showed 25 carbons; six quaternary, eight methines, eight methylenes and three methyl groups; the HSQC and ^13^C-NMR for **2** showed 25 carbons; six quaternary, nine methines, seven methylenes and three methyl groups. Like **1**, a combination of one-dimensional (1D) and 2D NMR indicated that **2** also consists of a heteroaromatic moiety (adenine), a pentose sugar and a sesquiterpene.

The structure of the sesquiterpene was fully elucidated based on its ^1^H and ^13^C-NMR data. Fifteen carbons (three quaternary carbons, three methyl groups, six methylenes, two methines, one oxymethine) were assigned to the sesquiterpene moiety. The NMR spectra of **2** were similar to **1** [17], with the exception of a secondary alcohol (δ_C_ 65.6, δ_H_ 3.94, 1H, m). ^1^H-^1^H correlation spectroscopy (COSY) correlations were observed between the oxymethine proton (δ_H_ 3.94, m) and two pairs of methylene protons H-1 (δ_H_ 1.27, 1H, t and 1.80, 1H, dd) and H-3 (δ_H_ 2.52, 1H, t and 2.41, 1H, m) indicated a partial structure of -CH_2_-CH(OH)-CH_2_-. Further COSY correlations of H-6 (δ_H_ 1.70, 1H, m and δ_H_ 1.38, 1H, t) to H-5 (δ_H_ 2.60, 1H, dd) and H-7 (δ_H_ 1.94, 1H, m), H-8 (δ_H_ 1.56, 1H, m and δ_H_ 1.38, 1H, t) to H-7 (δ_C_ 1.94, 1H, m) and H-9 (δ_H_ 1.52, 1H, m and δ_H_ 1.38, 1H, t) lead to formation of another partial structure of -CH-CH_2_-CH-CH_2_-CH_2_-. ^1^H-^13^C heteronuclear multiple bond correlation (spectroscopy) (HMBC) correlations between H-3 protons (δ_H_ 2.52 1H, t and 2.41, 1H, m) and C-15 (δ_C_ 22.4), C-1 (δ_C_ 50.8), C-2 (δ_C_ 65.6) and C-4 (δ_C_ 60.4) were observed. Further ^1^H-^13^C HMBC correlations were observed between (H-1 protons) δ_H_ 1.27 and 1.80 and C-14 (δ_C_ 20.7), C-3 (δ_C_ 46.6), C-10 (δ_C_ 35.6), C-2 (δ_C_ 65.6) and C-9 (δ_C_ 46.4). These enabled the assignment of the alcohol at C-2. Additional HMBC cross peaks between H-5 proton (δ_H_ 2.60) and C-1 (δ_C_ 50.8), C-4 (δ_C_ 60.4), C-10 (δ_C_ 35.6), C-14 (δ_C_ 20.7) and between H-14 methyl protons to C-10 (δ_C_ 35.6), C-9 (δ_C_ 46.4) and C-5 (δ_C_ 48.5) allowed the completion of the bicyclic ring. The vinyl signals (δ_C_ 109.0 (C-13), δ_H_ 4.65 (H-13) and δ_C_ 151.5 (C-11)) and a methyl group (δ_C_ 21.1, δ_H_ 1.68) corresponded to the sesquiterpene head in eudesmane-type sesquiterpenes. HMBC correlations between the H-7 proton (δ_H_ 1.94, 1H, m) and C-13 (δ_C_ 109.0), C-11 (δ_C_ 151.5) and C-12 (δ_C_ 21.1) confirmed the attachment of the isopropylene unit at C-7 of the sesquiterpene ring.

The relative stereochemistry at the asymmetric carbon centers at C-4, C-5, C-7, and C-10 was assigned by rotating frame nuclear Overhauser enhancement spectroscopy (ROESY) experiments. A number of cross-peaks at H-2β (δ 3.94)/H-1β (δ 1.80), H-2β/H-3β (δ 2.41), H-2β (δ 3.94)/H-14 (δ 1.06), H-2β/H-15 (δ 1.46), H-6β (δ 1.38)/H-8 (δ 1.56), H-6β/H-9 β (δ 1.52), H-6β/H-14, H-6β/H-15, H-8β (δ 1.56)/H-14 and H-14/H-15 were observed. These 1,3-diaxial correlations of H-14 and H-15 methyl protons and neighboring protons indicated that their orientation was axial. In contrast, other series of ROESY correlations at H-1α (δ 1.30)/H-3α (δ 2.52), H-1/H-5 (δ 2.60), H-1/H-9α (δ 1.38), H-3/H-5, H-5/H-6α (δ 1.70), H-5/H-7 (δ 1.94), H-5/H-12 (δ 1.68) and H-5/H-9α (δ 1.38) showed that these protons had an opposite orientation at the decalin plane. Thus, the relative configurations were assigned to be *trans* (C-5 and C-10) and 2*R*, 4*R*, 5*R*, 7*R*, 10*S* for the ring junction asymmetric carbons, respectively.

Assignment of the relative stereochemical configuration of the C-2 hydroxyl group was based on ROESY correlations observed between the H-2β proton (δ_H_ 3.94) and C-14 (δ_H_ 1.06) and C-15 (δ_H_ 1.46) methyl groups. The presence of two downfield aromatic protons at δ_H_ 8.24 (1H, s, H-2′) and δ_H_ 8.26 (1H, s, H-8′) suggested they were connected to nitrogen atoms. These two protons were HMBC-coupled to five downfield carbons δ_C_ 152.9 (C-2′), 148.8 (C-4′), 121.6 (C-5′), 155.7 (C-6′) and 141.2 (C-8′) confirmed the presence of the adenine base [19]. Long-range HMBC correlations between the C-15 methyl proton signals (δ_H_ 1.46) attached to C-4 (δ_C_ 60.4) of sesquiterpene and C-6′ (δ_C_ 155.7) of the adenine confirmed the position of linkage between the C-4 of eudesme-11-ne and the adenine base. The absence of any observable HMBC correlation between the C-15 methyl protons and the C-2′ carbon of the adenine confirms that the point of sesquiterpene attachment cannot be at the N-1′ position of adenine.

The ribofuranosyl was characterized by six proton signals in the region 3.74–5. 94 in the ^1^H-NMR spectrum. Based on COSY, HSQC and HMBC correlations, five carbons δ_C_ 91.3 (C-1″), 88.3 (C-4″), 75.4 (C-2″), 72.7 (C-3″), and 63.5 (C-5″) were assigned to the sugar moiety. A coupling constant of 6.5 Hz of the anomeric proton H-1″ (δ_H_ 5.94) and a larger magnitude (>2.15 ppm) in the difference between H-2′ (δ_H_ 8.24) and H-1″ (δ_H_ 5.94) protons indicated a β-anomer sugar [20]. The linkage between the adenine and ribose moiety was based on HMBC correlation between anomeric proton H-1″ (δ_H_ 5.94) and δ_C_ 148.8 (C-4′) and 141.2 (C-8′). ROESY correlations observed between the anomeric proton H-1″/H-4″, H-1″/H-2″, H-1″/H-3″ and between H-2″/H-3″ and H-2′/H-4′ suggest a *cis* conformation of the H-2″ and H-3″ hydroxyl groups. Comparison of the NMR data with the literature values suggested a d-ribose sugar [21]. In addition, the observed optical rotations of both compounds (**1** [α]^20^_D_ -76.92 (MeOH) and **2** [α]^20^_D_ -70.92 (MeOH) were negative as in the case of previously isolated **1** [α]^20^_D_ -78.7 [17]. Moreover, the circular dichroism (CD) spectrum of **2** shows two negative cotton effect bands at 260 and 215 nm (Figure S13), consistent with the CD spectrum from adenosine [22,23,24], which features, like most of the naturally-occurring monosaccharides, a d-configured ribose [22,23,24]. Finally, the subsequently identified biosynthetic origin of **1** and **2** strikingly suggests a d-configured ribose (see below).

### 2.2. Biological Activity of 2-Hydroxysorangiadenosine and Sorangiadenosine

The sesquiterpene–nucleoside compounds **1** and **2** showed biological activity against the Gram-positive bacteria *Bacillus subtilis* DSM10 and *Staphylococcus aureus* Newman (Table 1). **1** was cytotoxic towards HCT (human colon carcinoma cell line, DSMZ No. ACC 581) and KB3.1 cells at IC_50_ of 30.0 µg/mL and 39.46 µg/mL while **2** featured activity against HCT cell lines at IC_50_ of 52.0 µg/mL (Table 2). In conclusion, **1** shows better bioactivity compared to **2**. This may be due to the additional oxygen atom in **2**, which significantly increases the polarity of the sesquiterpene–nucleoside scaffold and limits the cell membrane penetration capability of **2** (a finding also reflected in the difference of the retention times).

The results of our bioactivity profiling of **1** (and **2**) resemble the findings of the previously observed antimicrobial range of **1** [17]. Despite the fact that former bioactivity screening of **1** displayed moderate bioactivity against the Gram-negative bacterium *Proteus vulgaris* ATCC 3851 (Minimum inhibitory concentration (MIC) at 6.25 µg/mL) and *Salmonella typhimurium* ATCC 14028 (MIC at 12.5 µg/mL) [17], the biological activity of these sesquiterpene–nucleosides seem to be focused on Gram-positive bacteria according to our findings. The lack of biological activity of **1** and **2** against *E. coli* test strains (*E. coli* wild type (WT) (DSM 1116) and *E. coli acrB* JW0451-2 see Table 1, *Escherichia coli* ATCC 25922 see Ahn et al. [17]) underlines this conclusion and highlights the difficulty of finding new compounds capable of, presumably, penetrating the outer membrane of Gram-negative bacteria [25,26].

### 2.3. Identification of the 2-Hydroxysorangiadenosine Biosynthetic Gene Cluster

According to retrobiosynthetic considerations, **1** and **2** are made of the building blocks adenosine (available from primary metabolism) and the eudesmane-type sesquiterpene, which can be generated from farnesyl pyrophosphate (FFP). Supplementation with sodium acetate ^13^C_2_—representing the starting precursor for the mevalonate pathway—did not result in a significant mass shift in the isotopic pattern of **1** and **2** (Figures S1 and S4). Therefore, the previously characterized myxobacterial l-leucine degradation pathway (including the formation of isovaleryl-coenzyme A (CoA), dimethylacyrlyl-CoA and 3-methylglutaconyl-CoA) [27] that branches from the mevalonate-dependent isoprenoid biosynthesis pathway might be involved in the formation of **1** and **2**. The observed mass shifts of +5 Da, +10 Da and +14 Da in the isotopic pattern of **1** and **2** upon dimethyl acrylic acid d_6_ supplementation suggest that the sesquiterpene scaffold originates from this myxobacterial isoprenoid pathway (Figures S2 and S5). In addition, genomic investigation of *V. cumulatum* MCy10943^T^ revealed the presence of all genes encoding the proteins which are required for the leucine degradation pathway [27] and the alternative biosynthesis of isovaleryl-CoA [28] (Figure S14 and Table S1). Corresponding mass shifts of + 4 Da and + 5 Da upon incorporation of adenosine monophosphate (^15^N_5_) (Figures S3 and S6) underline that adenosine is transferred as a purine nucleoside to the eudesmadiene scaffold rather than being generated de novo. In conclusion, these feeding experiments proved that **1** and **2** are most likely generated from the building blocks isopentenyl diphosphate (IPP) or dimethylallyl diphosphate (DMAPP) and adenosine.

**Figure 4:**
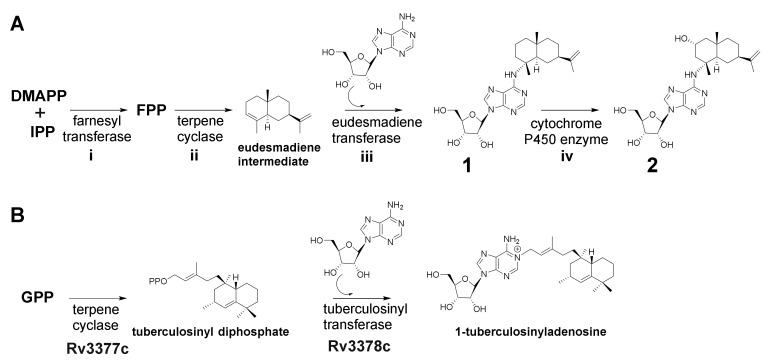
(**A**) Proposed biosynthetic route to **1** and **2** based on retrobiosynthetic considerations. (**B**) Biosynthesis of 1-tuberculosinyladenosine [32].

Based on the elucidated chemical structure of **1** and **2** along with the findings from feeding experiments, we propose both to be biosynthesized by farnesyl transferase (i), which produces FFP, a terpene cyclase (ii) which converts the generated FFP to a eudesmane-type sesquiterpene (eudesmadiene intermediate, see Figure S16) and a eudesmadiene transferase (iii) to transfer the adenosine building block onto the eudesmane-type sesquiterpene (Figure 4A). The mechanism to transfer adenosine, which is present in most microorganisms, might work via hydroamination (direct addition of ammonia or primary and secondary amines to non-activated alkenes and alkynes) or alkylation [29,30]. A last maturation step involves the hydroxylation of **1** leading to the herein described structure of **2**. Since most bacterial terpene biosynthetic pathways involve tailoring steps through cytochrome P450 enzyme hydroxylations [31], we assume that the last tailoring step in the biosynthesis of **2** might be catalyzed as well by a cytochrome P450 enzyme (iv) (Figure 4A). This assumption is also supported by the fact that the secondary metabolome of MCy10943^T^ shows no signal corresponding to the sum formula of a hydroxylated eudesmadiene intermediate or intermedeol. In addition, three secondary metabolites with the mass of 205 *m*/*z* and the sum formula C_15_H_25_ (which can be linked to non-hydroxylated eudesmadiene intermediates) are present in the crude extract of MCy10943^T^ (Figure S16).

In contrast to the first, second and last biosynthetic steps, the catalytic reaction of a specific eudesmadiene transferase is unprecedented. This biosynthetic enzyme would act differently from classical *cis*-prenyl transferases by catalyzing the condensation of eudesmadiene pyrophosphate and adenosine to generate the previously discovered structure of **1**. A similar reaction has been described for the biosynthesis of 1-tuberculosinyladenosine, where the second biosynthetic enzyme Rv3378c works as a tuberculosinyl transferase, catalyzing the condensation of tuberculosinyl pyrophosphate and adenosine to generate 1-tuberculosinyladenosine [32] (Figure 4B).

For this reason, genome sequence analysis was focused on gene clusters harboring genes encoding terpene or diterpene cyclases to identify the biosynthetic locus accounting for the production of **1** and **2**. MCy10943^T^ harbors, according to the antibiotics and secondary metabolite analysis shell (antiSMASH) [33], nine putative terpene gene clusters (Table 3). In-silico genome sequence analysis of several myxobacterial strains (including alternative producers of **1** and **2** and strains with BGCs homologous to three terpene gene clusters found in MCy10943^T^) in combination with metabolomic profiling of corresponding crude extracts facilitated the identification of a candidate biosynthetic gene cluster (SI 2.3).

**Figure 5:**
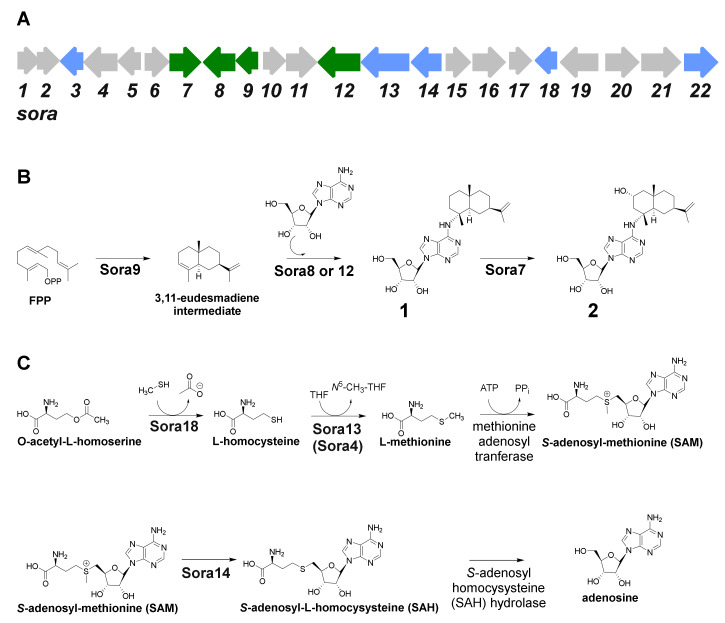
(**A**) Genetic organization of the candidate biosynthetic gene cluster responsible for the biosynthesis of sorangiadenosine (**1**) and 2-hydroxysorangiadenosine (**2**). (**B**) Proposed biosynthetic route leading to **1** and **2**. (**C**) Catalyzed reactions to increase the supply of adenosine.

The candidate terpene gene cluster, No. 1, harbors all genes necessary for the formation of **1** and **2** (Table 3). Terpene gene cluster No. 1 contains 22 open reading frames (*sora1–22*) and comprises 27.920 bp (Figure 5A, Table 4). The myxobacterial strains *Archangium violaceum* Cb vi76 [34] and *Archangium* sp. Cb g35 [35] feature similar biosynthetic gene cluster organizations according to antiSMASH evaluation (however, these gene clusters are missing *sora12*, see below). The gene *sora9* encodes a terpene cyclase, which could produce the eudesmane-type sesquiterpene building block, while *sora8* and *sora12* presumably catalyze the transfer of the adenosine onto the terpene scaffold (Figure 5B). Both transferases share high mutual sequence similarity and probably exert the same catalytic function. Only the 2-hydroxysorangiadenosine gene cluster in *V. cumulatum* MCy10943^T^ contains *sora12*, which might be the reason for the tremendously improved production in comparison to the identified alternative myxobacterial producers (SI 1.3).

The identified type I terpene cyclase Sora9 contains all typical sequence motifs, such as the aspartate-rich motif DDxxxD, the pyrophosphate sensor (R), the NSE triad and the RY dimer [42]. In addition, terpene gene cluster No. 1 harbors several genes responsible for adenosine supply. In prokaryotes, adenosine can be formed either by salvage pathways or via de-novo synthesis, which starts from simple primary metabolites and forms inositol monophosphate (IMP) as a branch-point intermediate to form further guanosine monophosphate (GMP) and adenosine monophosphate (AMP), of which the later can be converted to adenosine via adenosine/deoxycytidine kinase or by the catalytic action of 5′-nucleotidase. In addition, adenosine can also be produced in the context of l-homocysteine metabolism, where *S*-adenosyl-homocysteine (SAH) is converted to adenosine via the SAH hydrolase. The gene for the conversion of *O*-acetyl-homoserine to l-homocysteine via an *O*-acetylhomoserine sulfhydrylase (*sora18*) is located within the terpene gene cluster No. 1. The intermediate l-homocysteine can then subsequently be further converted by a vitamin B_12_-independent synthase (encoded by *sora4*) and a methionine synthase (encoded by *sora13*) to l-methionine. An ATP-dependent l-methionine adenosyltransferase catalyzes the production of *S*-adenosyl-methionine (SAM), which can be methylated by a SAM-dependent methyltransferase encoded by *sora14.* The catalytic product of Sora14, *S*-adenosyl-homocysteine (SAH), finally undergoes hydrolytic cleavage to yield adenosine, which can in turn be used as a building block for **1** and **2** (Figure 5C). These proteins further contribute to the availability of adenosine. In addition, these findings further support the assigned d-configuration of the ribose in **1** and **2** (see Section 2.1), since these genes are inseparably connected with the d-configured ribose, in particular the SAM-dependent methyltransferase Sora14. The truncated 2-hydroxysorangiadenosine BGC in the alternative producer *Cystobacter* sp. strain MCy9101 (SBCb004) also harbors *sora13, sora14* and *sora18* (Figure S18). Therefore, it seems likely that these encoded proteins are directly involved in adenosine supply for **1** and **2**. As shown for the biosynthesis of 1-tuberculosinyladenosine, the gene responsible for the transfer of the adenosine scaffold is closely located to the terpene cyclase gene [32]. However, it cannot be excluded that the genes responsible for the generation of adenosine are located elsewhere in the genome.

In conclusion, these findings strongly support our conclusion that the identified BGC is involved in the proposed biosynthetic route leading to the formation of **1** and **2**.

## 3. Discussion

The weak antibacterial myxobacterial sesquiterpene–nucleoside **1** was isolated from *S. cellulosum* KM1003 in 2008 [17]. Distinct structural features of **1**, such as the heteroaromatic adenosine, the pentose sugar d-ribofuranose and the bicyclic sesquiterpene of eudesmane type are of particular biosynthetic interest, since they have not been reported for other myxobacterial natural products and are rare among other producers. The biosynthetic pathway and gene cluster leading to the formation of **1** have remained elusive. Unlike naturally occurring nucleotides and nucleoside natural products and terpenes, there are only few terpene–nucleoside/nucleotide hybrids known up-to date, in particular from microorganisms such as 1-tuberculosinyladenosine produced by *Mycobacterium tuberculosis* [32] or 2-methylthio-*N*^6^-(Δ^2^-isopentenyl)adenosine from *E. coli* [43]. The cytokines are described as plant-derived terpene–purine derivatives involved in cell growth and differentiation and act complementary with auxin as plant growth hormones [44,45]. The zeatins (adenine-type cytokinins like lupinic acid [46]) feature a hydroxylated isopentenyl building block on the N-6 position of adenosine. Furthermore, the agelasines [47,48], agelasimines [49] and asmarines [50] are terpene–purines originating from different genera of sponges (*Agela* sp., *Raspailia* sp.) which feature a diterpene attachment on the N-6 (and N-7 in case of asmarines) position of adenine [51].

In this study, we report the isolation and structure elucidation of the new natural product **2** alongside with the known derivative **1** from the myxobacterium *V. cumulatum* MCy10943^T^. For the first time, we provide biosynthetic insights leading to the production of these unique sesquiterpene–adenosine hybrids in myxobacteria. The presented discovery and in silico biosynthetic study paves the way for further genetic investigation of this intriguing pathway and the underlying production of these unique bioactive chemical scaffolds.

The development of genetic tools for the myxobacterium *V. cumulatum* MCy10943^T^—which can be a difficult endeavor for myxobacterial strains [52]—would allow experimental correlation between the identified BGC and the production of **1** and **2**. After genetic confirmation, the key biosynthetic genes could be heterologously expressed in the myxobacterial model host *M. xanthus* DK1622 in order to genetically engineer the biosynthetic pathway for an improved production rate or streamline the generation of new derivatives. In-depth biochemical investigation of the biosynthesis of **1** and **2** would be attained through recombinantly generated biosynthetic proteins to uncover single biosynthetic reactions or reconstitute the biosynthesis in vitro.

In conclusion, the presented discovery and the hypothesized biosynthetic pathway of **1** and **2** are essential prerequisites for further biosynthetic studies, production optimization and the generation of new sorangiadenosine derivatives.

## 4. Materials and Methods

### 4.1. Applied Software, Sequence Analysis and Bioinformatics Methods

DNA was isolated using a Qiagen Genomic-tip 100/G (Qiagen, Hilden, Germany) according to the instructions of the manufacturer. A SMRTbell™ template library was prepared according to the instructions from Pacific Biosciences (Pacific Biosciences, Menlo Park, CA, USA), following the Procedure and Checklist—Greater Than 10 kb Template Preparation. Briefly, for preparation of 15 kb libraries, 8 µg genomic DNA was sheared using g-tubes™ from Covaris (Covaris, Woburn, MA, USA), according to the manufacturer’s instructions. DNA was end-repaired and ligated overnight to hairpin adapters applying components from the DNA/Polymerase Binding Kit P6 from Pacific Biosciences (Pacific Biosciences, Menlo Park, CA, USA). Reactions were carried out according to manufacturer’s instructions. BluePippin™ Size-Selection to greater than 7 kb was performed according to the manufacturer’s instructions (Sage Science, Beverly, MA, USA). Conditions for annealing of sequencing primers and binding of polymerase to purified SMRTbell™ template were assessed with the Calculator in RS Remote, Pacific Biosciences (Pacific Biosciences, Menlo Park, CA, USA). Two SMRT cells were sequenced on the PacBio *RSII* (Pacific Biosciences, Menlo Park, CA, USA), taking 240 min movies.

The neutral sum formulas of the identified two target masses (C_25_H_37_N_5_O_4_ corresponding to **1** and C_25_H_37_N_5_O_5_ corresponding to **2**) were used to search in several chemical structure databases (SciFinder, Dictionary of Natural Products, ChemSpider, PubChem, ChEMBL, ZINC and Super Natural II). The *Vitiosangium cumulatum* MCy10943^T^ genome was screened for secondary metabolite BGCs using the antiSMASH 5.0 [33] online tool and the software Geneious Prime^®^ (Biomatters Ltd., Auckland, New Zealand, 2020.0.5) [53]. The nucleotide or amino acid sequence of interest was aligned with the basic local alignment search tool (BLAST) against our in-house genome database or the publicly available nucleotide database, in order to find homologous genes or proteins. The functional prediction of ORFs was performed by either using protein blast and/or blastx programs and Pfam [54]. The nucleotide sequence of the 2-hydroxysorangiadenosine BGC originating from MCy10943^T^ has been deposited in GenBank and is accessible under the accession number MT520811. The same nucleotide sequence will be implemented in the Minimum Information about a Biosynthetic Gene cluster (MIBiG) database. Further information concerning gene sequences can be found in the Supplementary Information.

### 4.2. Maintenance of Bacterial Cultures and Feeding Experiments with Stable Isotope-Labeled Building Blocks

*Vitiosangium cumulatum* MCy10943^T^ was routinely cultivated at 30 °C in CyHv3 medium or agar plates (0.2% soytone (BD), 0.3% casitone (BD), 0.2% glucose, 0.8% soluble starch (Roth), 0.15% yeast extract (BD), 0.1% CaCl_2_ × 2H_2_O, 0.1% MgSO_4_ × 7H_2_O, 50 mM HEPES (for agar plate cultures 15 g/L agar) adjusted pH to 7.2 with 10N KOH before autoclaving, added after autoclaving 8 mg/L Fe-EDTA). Liquid cultures were grown in Erlenmeyer flasks on an orbital shaker at 180 rpm for 3–6 days. Feeding experiments were performed by cultivating the strain in 20 mL CyHv3 broth using 100 µL inoculum (0.5% inoculum volume). The cultures were supplemented with 1 mL (*v*/*v*) sterile amberlite resin XAD-16 (Sigma-Aldrich Chemie GmbH, Taufkirchen, Germany) and fed for five consecutive days with 20 μL of a 0.1M solution of either sodium acetate (^13^C_2_), sodium dimethyl acrylic acid (d_6_) or adenosine monophosphate (^15^N_5_) at 30 °C, at 180 rpm. The combined cells and resin were harvested by centrifugation after seven days of incubation before extraction. The supernatant was discarded, whereas the combined cells and resin were extracted with a mixture of 25 mL methanol (MeOH) and 25 mL acetone, stirred for 2 h and filtered through filter paper, and the solvent of the extracts was removed under vacuum. The re-dissolved extracts (2 mL) were diluted with MeOH (1:3 (extract/MeOH, *v*/*v*) and centrifuged, and the supernatant was subjected to HPLC-MS analysis as described further below.

### 4.3. Analysis of Secondary Metabolism of Broth Extracts

The secondary metabolism of broth extracts was analyzed by high-performance liquid chromatography–high-resolution electrospray ionization-diode array-detector–mass spectrometry (HPLC-HRESI-DAD-MS) on a maXis 4G mass spectrometer (Bruker Daltonics, Billerica, MA, USA) coupled with a Dionex UltiMate 3000 Rapid Separation (RS)LC system (Thermo Fisher Scientific, Waltham, MA, USA) using a BEH C18 column (100 × 2.1 mm, 1.7 μm) (Waters, Eschborn, Germany) with a gradient of 5–95% acetonitrile (ACN) + 0.1% formic acid (FA) in H_2_O + 0.1% FA at 0.6 mL/min and 45 °C over 18 min with ultraviolet (UV) detection by a DAD at 200–600 nm. Mass spectra were acquired from 150 to 2000 *m*/*z* at 2 Hz. Detection was performed in the positive MS mode. The plugin for Chromeleon Xpress (Thermo Fisher Scientific, Waltham, MA, USA, version 6.8) was used for operation of the Dionex UltiMate 3000 RSLC system. HyStar (Bruker Daltonics, Billerica, MA, USA, version 3.2) was used to operate on the maXis 4G mass spectrometer system. HPLC-MS mass spectra were analyzed with DataAnalysis (Bruker Daltonics, Billerica, MA, USA, version 4.2).

### 4.4. Compound Isolation

At the end of fermentation (60 L), wet cell mass and adsorbent resin XAD-16 were harvested by centrifugation and extracted three times with 2 L of acetone. The extract was dried under vacuum resulting in 7.68 g of crude extract. The crude extract was dissolved in 2 L MeOH and partitioned using 2 L hexane solvent. The MeOH layer was dried (5.34 g) and dissolved in 2 L deionized water followed by further partitioning using chloroform (CHCl_3_). The CHCl_3_ layer was dried under vacuum to yield 3.95 g of extract. The 3.95 g of CHCl_3_ extract was subjected to flash chromatography on a Isolera™One (Biotage, Uppsala, Sweden) with a SNAP 100 g column packed with silica gel (60 Å, 70–230 mesh, 63–200 μm), using 100% *n*-hexane (5 column volume (CV)), 95% *n*-hexane /ethyl acetate (EA) (5 CV), 95% *n*-hexane/EA to 100% EA (25 CV), 100% EA (5 CV), 95% EA /MeOH gradient as eluents. The flow rate was 50 mL/min while the UV/Vis absorption was set at 260 nm and 320 nm. Fractions of 45 mL were collected and measured on a Dionex UltiMate 3000 RSLC system (Thermo Fisher Scientific, Waltham, MA, USA) coupled to an amaZon ion trap MS (Bruker Daltonics, Billerica, MA, USA) using a BEH C18, 100 × 2.1 mm, 1.7 μm dp column equipped with a C18 precolumn (Waters, Eschborn, Germany) to detect the fractions containing **1** or **2**. Fractions 83–95 were pooled and dried under vacuum to yield 1.23 g extract. Purification of sample was done using an UltiMate 3000 semi-preparative system coupled to a Thermo Scientific Dionex UltiMate 3000 Series automated fraction collector (Bruker Daltonics, Billerica, MA, USA). Separation was performed on a C18 Phenomenex Luna (100 Å, 5 μm, 10 × 250 mm) LC column (Phenomenex, Torrance, CA, USA) and eluted with water (0.1% FA) as solvent **A** and ACN (0.1% FA) as solvent **B** at a flow rate of 5 mL/min. The initial gradient was held at 5% ACN for 2 min and then elevated to 60% ACN within 5 min. This was followed by an increase from 61% to 70% ACN during a period of 25 min and then to 95% ACN within 5 min. The gradient was held at 95% ACN for 2 min and then ramped back to 5% ACN over 1 min. The column was re-equilibrated at 5% ACN for 5 min. Detection of the sesquiterpene–nucleosides was facilitated via mass spectrometry on the Agilent 1100 series (Agilent Technologies, Santa Clara, CA, USA) coupled to the HCT 3D ion trap (Bruker Daltonics, Billerica, MA, USA) or with a UV detector on the Dionex UltiMate 3000 RSLC system by UV absorption at 220 nm, 260 nm, 320 nm and 400 nm. The pure compounds were subsequently dried by lyophilization and resulted in 4.91 mg of compound **2** and 3.88 mg of compound **1** at a retention time of 18.23 min and 22.14 min, respectively.

### 4.5. NMR-Based Structure Elucidation, Chiroptical and CD Measurement

The chemical structures of **1** and **2** were determined via multidimensional NMR analysis. ^1^H-NMR, ^13^C-NMR and 2D spectra were recorded at 500 MHz (1H)/175 MHz (^13^C), conducting an Ascend 500 spectrometer using a cryogenically cooled triple resonance probe (Bruker Biospin, Rheinstetten, Germany). Samples were dissolved in methanol-d_4_. Chemical shifts are reported in ppm relative to tetramethylsilane; the solvent was used as the internal standard.

Chiroptical measurements of **1** and **2** in MeOH ([α]_D_) were obtained on a Model 341 polarimeter (PerkinElmer Inc., Waltham, MA, USA) in a 100 × 2 mm cell at 20 °C. Circular dichroism measurements were performed for **2** at 1.0 mM in MeOH (190–400 nm) with the J-1500 CD spectrophotometer (JASCO, Easton, MD, USA).

### 4.6. Bioactivity Profiling

Standard sterile microbiological techniques were maintained throughout. All microorganisms and cell lines were handled according to standard procedures and were obtained from the German Collection of Microorganisms and Cell Cultures (Deutsche Sammlung für Mikroorganismen und Zellkulturen, DSMZ) or were part of our internal strain collection and were cultured under conditions recommended by the depositor. Both **1** and **2** were tested in microbroth dilution assays on the following panel of microorganisms: *Escherichia coli* WT (DSM 1116), *E. coli* JW0451-2 (*acrB*-efflux pump deletion mutant of *E. coli* BW25113), *Pseudomonas aeruginosa* PA14, *Bacillus subtilis* DSM 10, *Mycobacterium smegmatis* MC^2^-155 (DSM 43756), *Staphylococcus aureus* Newman, *Candida albicans* DSM 1665, *Citrobacter freundii* DSM 30039, *Pichia anomala* DSM 6766 and *Acinetobacter baumannii* DSM 30007. For microbroth dilution assays, overnight cultures were prepared from cryogenically preserved cultures and were diluted to achieve a final inoculum of 10^4^–10^5^ colony-forming units (cfu)/mL. Serial dilutions of compounds were prepared in sterile 96-well plates in the respective test medium. The cell suspension was added and microorganisms were grown for 18–48 h at either 30 °C or 37 °C. Growth inhibition was evaluated by visual inspection, and given MIC values are the lowest concentrations of antibiotic at which no visible growth was observed.

To evaluate the cytotoxic capabilities of **1** and **2**, HCT-116 (human colon carcinoma cell line, DSMZ No. ACC 581) and KB-3-1 (cervix carcinoma cell line, DSMZ No. ACC 158) cells were seeded at 6 × 10^3^ cells per well of 96-well plates in 180 μL complete medium and treated with **1** or **2** in serial dilution after 2 h equilibration. After five days of incubation, 20 μL of 5 mg/mL MTT (thiazolyl blue tetrazolium bromide) in phosphate-buffered saline (PBS) was added per well and it was further incubated for 2 h at 37 °C. The medium was discarded, and cells were washed with 100 μL PBS before adding 100 μL isopropanol/10 N HCl (250:1) in order to dissolve formazan granules. The absorbance at 570 nm was measured using the microplate reader Infinite^®^ M200Pro (Tecan Group Ltd., Männedorf, Switzerland), and cell viability was expressed as a percentage relative to the respective MeOH control. IC_50_ values were determined by sigmoidal curve fitting.

## Figures and Tables

**Table 1:** Minimum inhibitory concentration (MIC) values of sorangiadenosine and 2-hydroxysorangiadenosine (**1** and **2**) against common microbial pathogens.

Microorganism	MIC (µg/mL) of Sorangiadenosine (1)	MIC (µg/mL) of 2-hydroxysorangiadenosine (2)
*B. subtilis* DSM 10	16	64
*E. coli* WT (DSM 1116)	>128	>128
*E. coli acrB* JW0451-2	>128	>128
*P. aeruginosa* PA14	>128	>128
*S. aureus* Newman	32	128
*C. freundii* DSM 30039	>128	>128
*A. baumanii* DSM 30007	>128	>128
*M. hiemalis* DSM 2656	128	>128
*P. anomala* DSM 6766	>128	>128
*M. smegmatis* MC^2^ 155	>128	>128
*C. albicans* DSM 1665	128	>128

**Table 2:** Half-maximal inhibitory concentrations (IC_50_ values in µg/mL) of sorangiadenosine, 2-hydroxysorangiadenosine (**1** and **2**) and doxorubicin (used as cytotoxic positive control) against HCT-116 (human colon carcinoma cell line, DSMZ No. ACC 581) and KB-3-1 (cervix carcinoma cell line, DSMZ No. ACC 158).

Cancer Cell Line	IC_50_ (µg/mL) of Sorangiadenosine 1	IC_50_ (µg/mL) of 2-hydroxysorangiadenosine 2	IC_50_ (µg/mL) of Doxirubicin
HCT-116	30.00	52.00	0.6
KB-3-1	39.46	>111.1	0.09

**Table 3:** Biosynthetic gene clusters identified through antiSMASH analysis harboring a terpene cyclase.

No.	Gene Cluster	Size (bp)	Location	Terpene Cyclase	Associated Biosynthesis ^1^
1	Terpene	27920	653776–674051	1 × Type I, 2 × Type II	This study
2	Terpene	42274	1269631–1311904	1 × Type I	Geosmin [36,37]
3	Terpene	41089	3415277–3456365	1 × Type I	Genome-Metab
4	Terpene	41071	3447909–3488979	1 × Type I	
5	Terpene	40978	3603511–3644488	1 × Type I	Genome-Metab
6	Terpene	41041	4852259–4893299	1 × Type I	Genome-Metab
7	Terpene/Type_III_PKS	69038	7156603–7225640	1 × Type II	Carotenoid [38,39,40,41]
8	Terpene/TfuA-rel.	50108	8404785–8454892	1 × Type I	
9	Terpene/RiPP	78053	12603875–12681927	1 × Type I	Geosmin [36,37]

^1^ Genome-Metab.; these gene clusters have been excluded to be responsible for the formation of **1** and **2**, due to the genome–metabolome correlation (myxobacterial strains with homologues BGCs featured no detectable production of **1** or **2**, see SI).

**Table 4:** Predicted functions of the proteins encoded by the (2-hydroxy)sorangiadenosine biosynthetic gene cluster.

Gene	Size (aa)	Proposed Function	Closest Homologue	Coverage/Similarity (%)
*sora1*	378	Oxidoreductase	WP_108075222	100/95.99
*sora2*	72	Hypothetical protein	WP_108075223	100/93.90
*sora3*	173	Hypothetical protein	WP_108075224	100/82.92
*sora4*	364	Methionine synthase (MetE)	WP_108075225	100/95.87
*sora5*	304	Hydrolase	WP_108075226	100/90.43
*sora6*	284	Hypothetical protein	WP_108075227	95/92.25
*sora7*	457	Cytochrome P450 enzyme	WP_108069092	98/95.67
*sora8*	518	Eudesmadiene transferase	WP_047856205	99/80.31
*sora9*	333	Terpene cyclase	WP_073564250	99.78/79.09
*sora10*	387	Hypothetical protein	WP_095976240	91/56.91
*sora11*	609	Sensory transducer	WP_146210122	92/74.69
*sora12*	509	Eudesmadiene transferase	WP_108075230	100/73.80
*sora13*	1171	Methionine synthase (MetH)	WP_108075232	100/97.61
*sora14*	299	SAM-dependent methyltransferase	WP_108075233	100/97.32
*sora15*	292	Patatin lipid acyl hydrolases	WP_108075234	100/98.97
*sora16*	375	Dehydrogenase	WP_052519033	91/82.11
*sora17*	205	Tet^R^ transcriptional regulator	WP_073564266	100/79.62
*sora18*	147	*O*-acetylhomoserine sulfhydrylase	WP_108075236	100/97.26
*sora19*	302	Hypothetical protein	WP_108075237	100/88.70
*sora20*	449	Thioredoxin	WP_140874099	99/54.81
*sora21*	279	Hypothetical protein	WP_158079939	100/77.34
*sora22*	630	Phosphotransferase	WP_073564278	100/79.83

## Supplementary Materials

The following are available online, Figure S1: Partial ESI + MS spectra for sorangiadenosine (**1**) supplemented with sodium acetate (^13^C_2_) (bottom) and culture broth without precursor feeding as control (top). Figure S2: Partial ESI + MS spectra for sorangiadenosine (**1**) supplemented with sodium dimethyl acrylic acid (d_6_) (bottom) and culture broth without precursor feeding as control (top). Figure S3: Partial ESI + MS spectra for sorangiadenosine (**1**) supplemented with adenosine monophosphate (^15^N_5_) (bottom) and culture broth without precursor feeding as control (top). Figure S4: Partial ESI + MS spectra for 2-hydroxysorangiadenosine (**2**) supplemented with sodium acetate (^13^C_2_) (bottom) and culture broth without precursor feeding as control (top). Figure S5: Partial ESI + MS spectra for 2-hydroxysorangiadenosine (**2**) supplemented with sodium dimethyl acrylic acid (d_6_) (bottom) and culture broth without precursor feeding as control (top). Figure S6: Partial ESI + MS spectra for 2-hydroxysorangiadenosine (**2**) supplemented with adenosine monophosphate (^15^N_5_) (bottom) and culture broth without precursor feeding as control (top). Figure S7: Partial ESI + MS spectra for sorangiadenosine (**1**). Figure S8: Partial ESI + MS spectra for 2-hydroxysorangiadenosine (**2**). Figure S9: HPLC-MS EICs of crude extracts of *Cystobacter* sp. MCy9101 (top) and *Vitiosangium cumulatum* MCy10943^T^ (bottom). EIC: Extracted ion chromatogram, green: 488.2873 *m*/*z*, with a width of 7.9 ppm, 2-hydroxysorangiadenosine [M + H]^+^. Figure S10: HPLC-MS EICs of crude extracts of *Cystobacter* sp. MCy9101 (top) and *Vitiosangium cumulatum* MCy10943^T^ (bottom). EIC: Extracted ion chromatogram, blue: 472.2924 *m*/*z*, with a width of 7.9 ppm, sorangiadenosine [M + H]^+^. Figure S11: HPLC-MS EICs of crude extracts of *Vitiosangium cumulatum* MCy10943^T^, *Cystobacter fuscus* DSM 2262, *Cystobacter fuscus* Cb fe15 and *Cystobacter ferrugineus* Cb fe23. EIC: Extracted ion chromatogram, green: 488.2873 *m*/*z*, with a width of 7.9 ppm, 2-hydroxysorangiadenosine [M + H]^+^. Figure S12: HPLC-MS EICs of crude extracts of *Vitiosangium cumulatum* MCy10943^T^, *Cystobacter fuscus* DSM 2262, *Cystobacter fuscus* Cb fe15 and *Cystobacter ferrugineus* Cb fe23. EIC: Extracted ion chromatogram, green: 472.2924 *m*/*z*, with a width of 7.9 ppm, sorangiadenosine [M + H]^+^. Figure S13: CD spectrum of 2-hydroxysorangiadenosine at 1.0 mM in MeOH in the area 190–400 nm, shows two negative cotton effect bands at 215 nm and 260 nm (top). Figure S14: Leucine degradation pathway (grey boxes) and the alternative biosynthesis of isovaleryl CoA observed in *Myxococcus xanthus* DK1622. Proteins are listed in Table S1. Figure S15: 1,10-cyclization and 1,11-cyclization from farnesylpyrophosphate (FFP) yield the (*E*,*E*)-germacrenyl cation, which is the proposed precursor for the generation of eudesmane-type sesquiterpene required for the biosynthesis of **1** and **2** or (*E*,*E*)-humulyl cation, respectively. Both enantiomers of the (*E*,*E*)-germacrenyl cation can be formed. (**B**) Isomerization mechanism of FFP to nerolidyl diphosphate (NPP). (**C**) NPP can be cyclized to a bisabolyl cation via 1,6-cyclization, to the cycloheptenyl cation via 1,7-cyclization, to the (*E,Z*)-germacrenyl cation via a 1,10-cyclization or to the (*E*,*Z*)-humulyl cation via a 1,11-cyclization. Both enantiomeric products of the 1,6-cyclization, 1,10-cyclization and 1,11-cyclization can be generated. Figure S16: (**A**) Proposed start of the sesquiterpene part during the biosynthesis of sorangiadenosine yielding the intermediate germacrenyl-cation. (**B**) Possible formation of intermedeol from the intermediate germacrenyl-cation. (**C**) Proposed formation of the different eudesmadiene building blocks. Figure S17: HPLC-MS EIC of *Vitiosangium cumulatum* MCy10943^T^ crude extract. EIC: Extracted ion chromatogram, black: 205.1956 *m*/*z*, with a width of 7.9 ppm, proposed non-hydroxylated eudesmadiene intermediates [M + H]^+^. Figure S18: Genetic organization of the truncated 2-hydroxysorangiadenosine BGC from *Cystobacter sp.* strain MCy9101 (SBCb004). Figure S19: Chemical structure and numbering of sorangiadenosine (**1**) and 2-hydroxysorangiadenosine (**2**). Figure S20: ^1^H-NMR spectrum of sorangiadenosine (**1**) in methanol-d_4._ Figure S21: ^13^C-NMR spectrum of sorangiadenosine (**1**) in methanol-d_4_. Figure S22: HSQC spectrum of sorangiadenosine (**1**) in methanol-d_4_. Figure S23: DQF-COSY spectrum of sorangiadenosine (**1**) in methanol-d_4_. Figure S24: HMBC spectrum of sorangiadenosine (**1**) in methanol-d_4_. Figure S25: ^1^H NMR spectrum of 2-hydroxysorangiadenosine (**2**) in methanol-d_4_. Figure S26: ^13^C NMR spectrum of 2-hydroxysorangiadenosine (**2**) in methanol-d_4_. Figure S27: HSQC spectrum of 2-hydroxysorangiadenosine (**2**) in methanol-d_4_. Figure S28: DQF-COSY spectrum of 2-hydroxysorangiadenosine (**2**) in methanol-d_4_. Figure S29: HMBC spectrum of 2-hydroxysorangiadenosine (**2**) in methanol-d_4_. Figure S30: ROESYphpr spectrum No. 1 of 2-hydroxysorangiadenosine (**2**) in methanol-d_4_. Figure S31: ROESYphpr spectrum No. 2 of 2-hydroxysorangiadenosine (**2**) in methanol-d_4_. Figure S32: ROESYphpr spectrum 3 of 2-hydroxysorangiadenosine (**2**) in methanol-d_4_. Figure S33: ROESYphpr spectrum 4 (expanded region) of 2-hydroxysorangiadenosine (**2**) in methanol-d_4_. Table S1: Proteins involved in leucine degradation and in the alternative isovaleryl-CoA biosynthesis in *Myxococcus xanthus* DK1622 and their identified homologues in *Vitiosangium cumulatum* MCy10943^T^. Table S2: NMR spectroscopic data for sorangiadenosine (**1**) in methanol-d_4_. Table S3: NMR spectroscopic data for 2-hydroxysorangiadenosine (**2**) methanol-d_4_.
