# Supplementary material for: 2-Hydroxysorangiadenosine: Structure and Biosynthesis of a Myxobacterial Sesquiterpene–Nucleoside
Source: Molecules. 2020 Jun 9;25(11):2676. doi: 10.3390/molecules25112676 (PMC7321100; doi:10.3390/molecules25112676)
Supplement: Supplementary file 1 [file molecules-25-02676-s001.pdf]

molecules

Supplementary Information

2-Hydroxysorangiadenosine: Structure and Biosynthesis of a Myxobacterial Sesquiterpene-Nucleoside

Dorothy A. Okoth [1,2], Joachim J. Hug [1,2], Ronald Garcia [1,2], Cathrin Spröer [3], Jörg Overmann [2,3] and Rolf Müller [1,2*]

1 Department Microbial Natural Products, Helmholtz-Institute for Pharmaceutical Research Saarland (HIPS), Helmholtz Centre for Infection Research (HZI) and Department of Pharmacy, Saarland University, Campus E8 1, 66123 Saarbrücken, Germany

2 German Center for Infection Research (DZIF), Partner Site Hannover-Braunschweig, Germany

3 Leibniz Institute DSMZ-German Collection of Microorganisms and Cell Cultures, Braunschweig, Germany

* Correspondence: rolf.mueller@helmholtz-hips.de; Tel.: +49-681-98806-3000

Table of Contents

1. Partial LC-MS spectra 3

1.1 Feeding experiments 3

1.2 Preparative LC-MS spectra 6

1.3 Alternative producer 8

1.4 CD spectrum 11

2. Biosynthetic *in silico* investigation 12

2.1 Leucine degradation (and alternative isovaleryl CoA biosynthesis) 12

2.2 Formation of sesquiterpene scaffolds and eudesmane-type sesquiterpenes 16

2.3 Metabolome-genome correlation 20

3. Structure elucidation 23

3.1 NMR spectroscopic data 23

3.2 ^{1}H and ^{13}C NMR spectra of sorangiadenosine and 2-hydroxysorangiadenosine 26

4. References 33

1. Partial LC-MS spectra

1.1 Feeding experiments

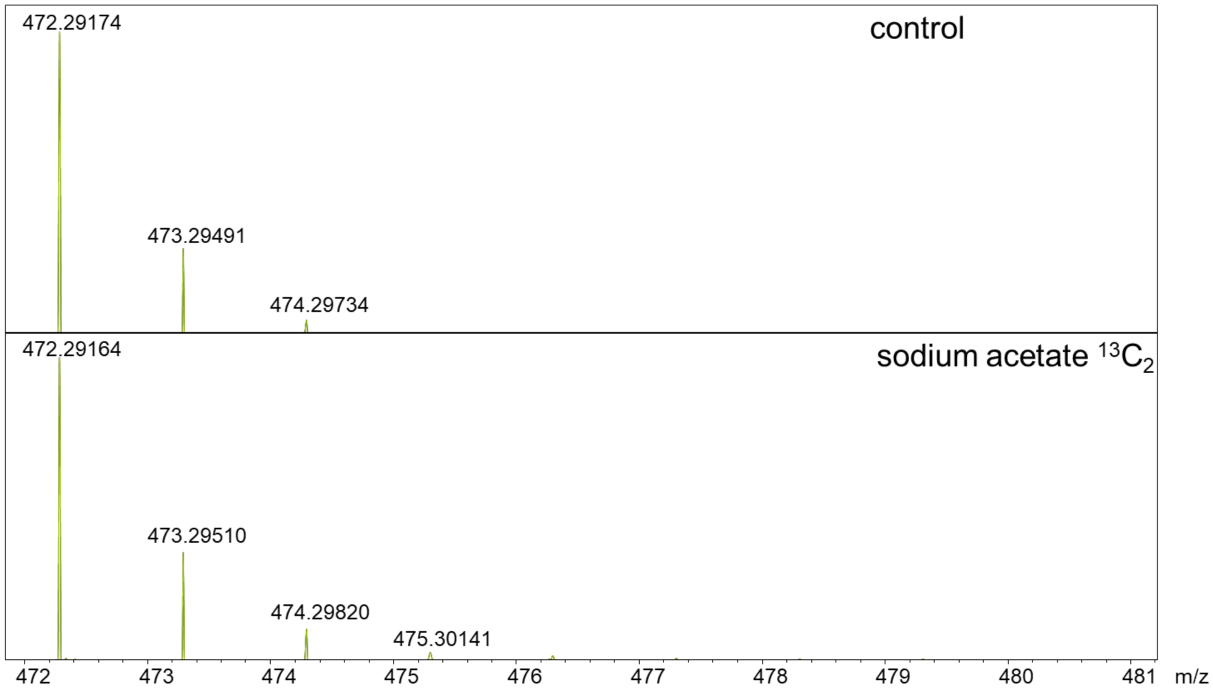

Figure S1. Partial ESI+MS spectra for sorangiadenosine (**1**) supplemented with sodium acetate ($^{13}C_2$) (bottom) and culture broth without precursor feeding as control (top).

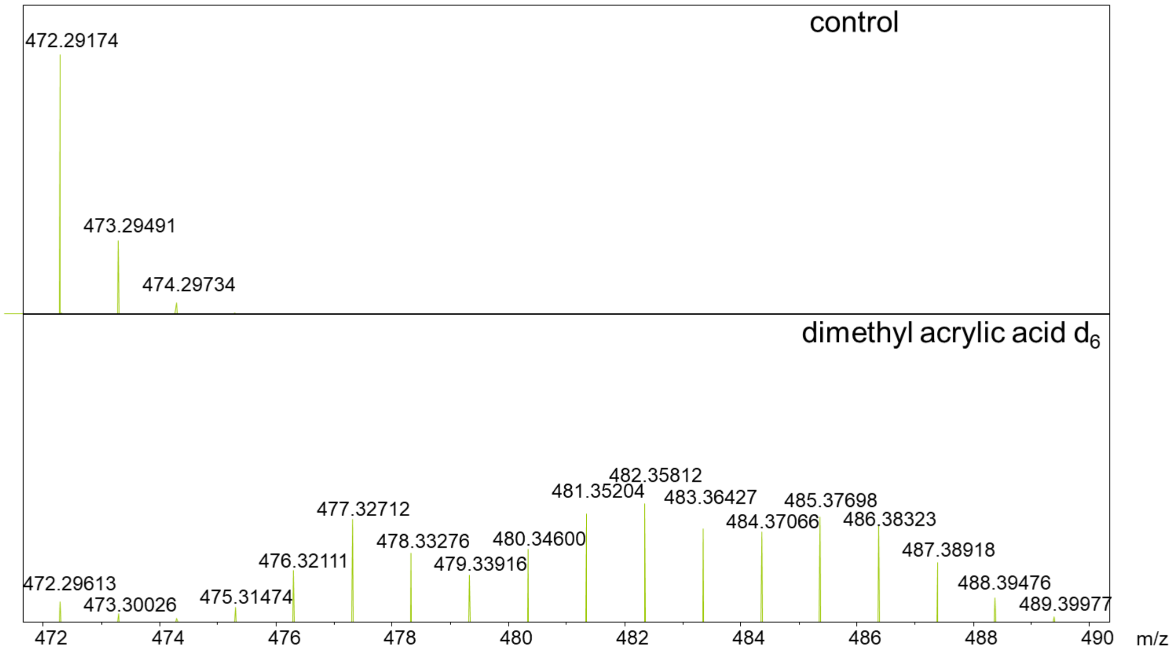

Figure S2. Partial ESI+MS spectra for sorangiadenosine (**1**) supplemented with sodium dimethyl acrylic acid (d_6) (bottom) and culture broth without precursor feeding as control (top).

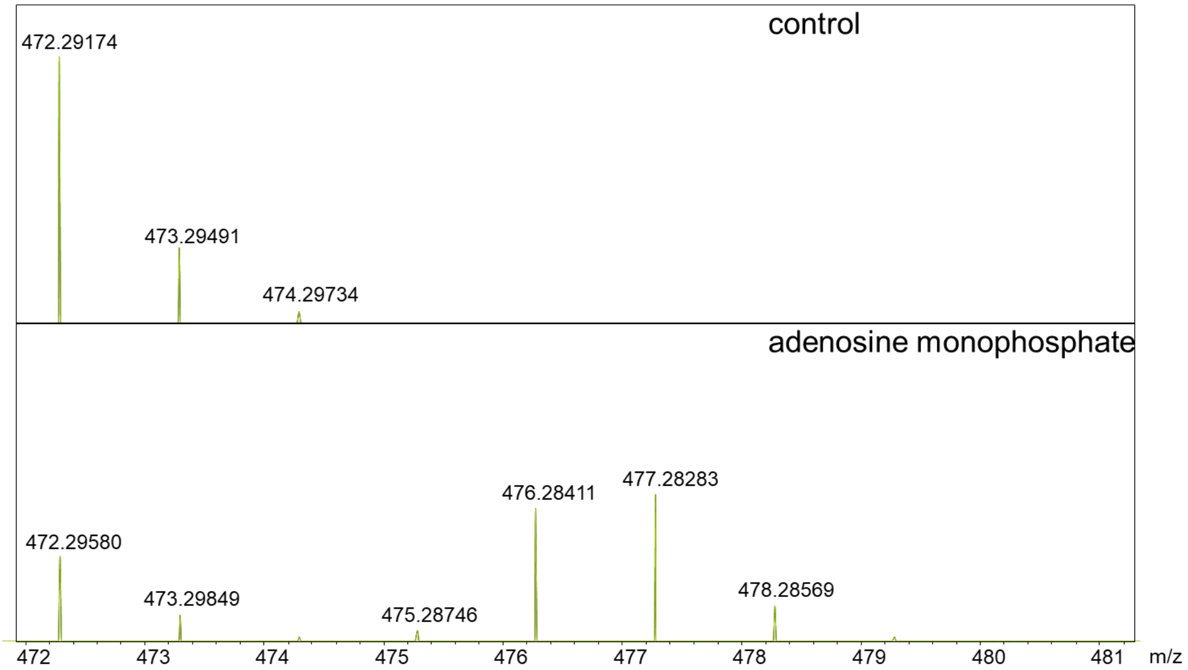

Figure S3. Partial ESI+MS spectra for sorangiadenosine (**1**) supplemented with adenosine monophosphate ($^{15}N_5$) (bottom) and culture broth without precursor feeding as control (top).

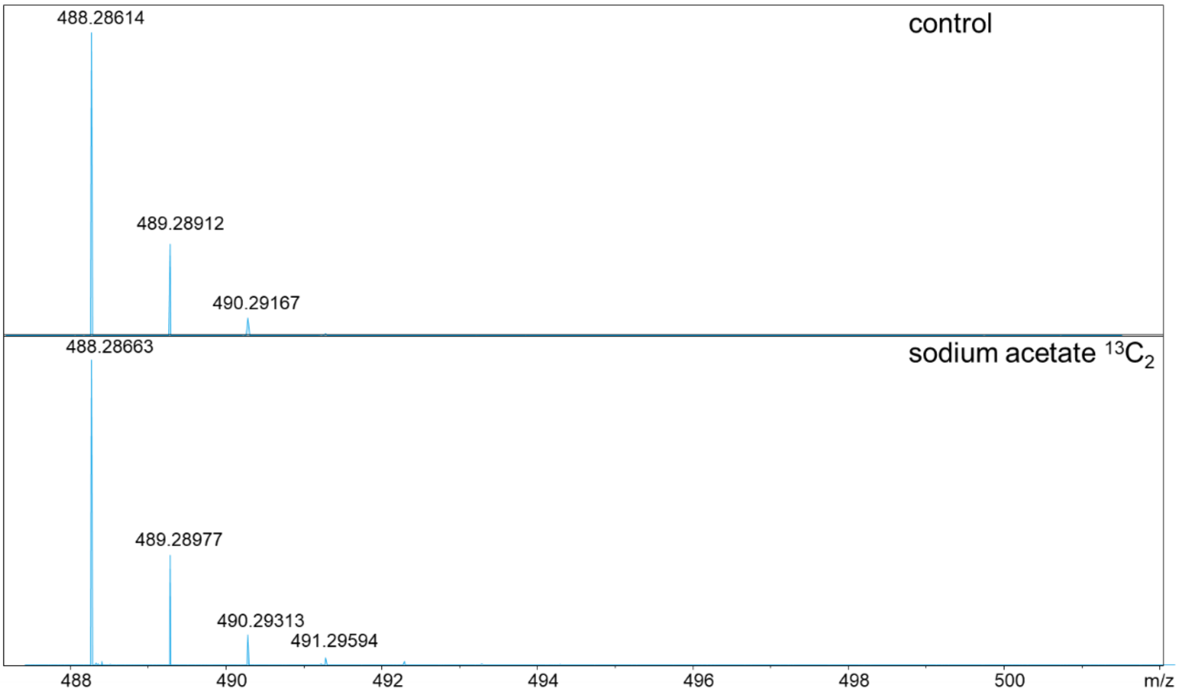

Figure S4. Partial ESI+MS spectra for 2-hydroxysorangiadenosine (**2**) supplemented with sodium acetate ($^{13}C_2$) (bottom) and culture broth without precursor feeding as control (top).

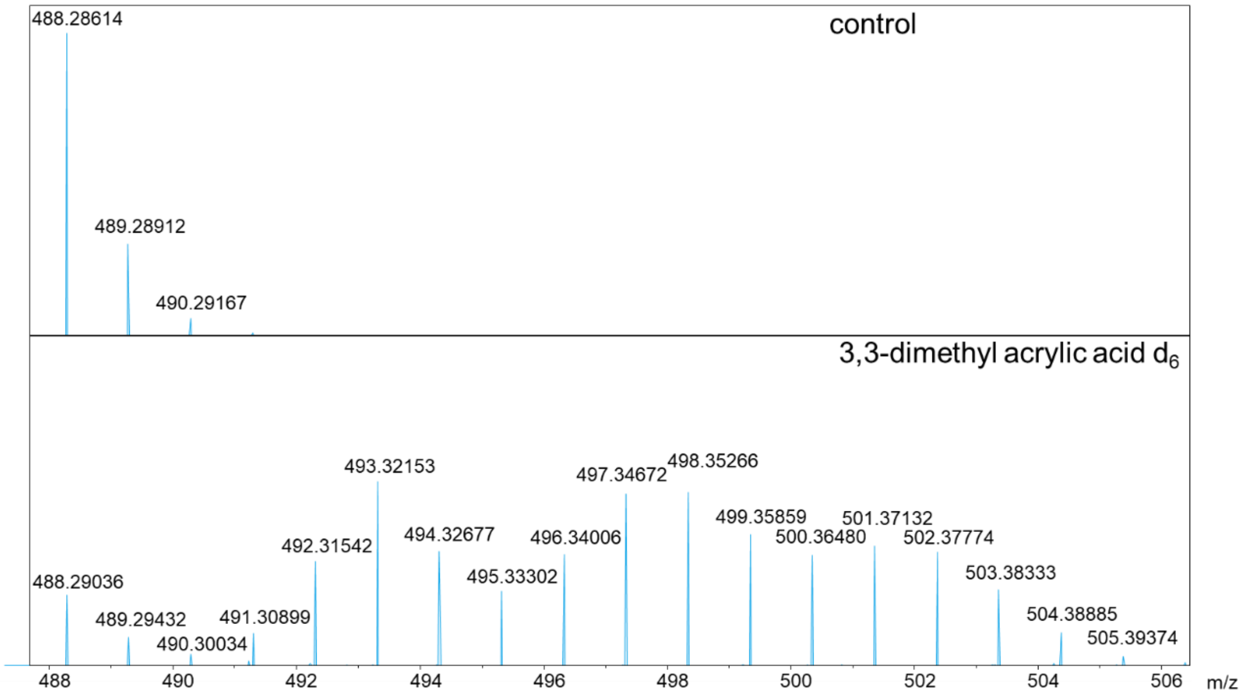

Figure S5. Partial ESI+MS spectra for 2-hydroxysorangiadenosine (**2**) supplemented with sodium dimethyl acrylic acid (d_6) (bottom) and culture broth without precursor feeding as control (top).

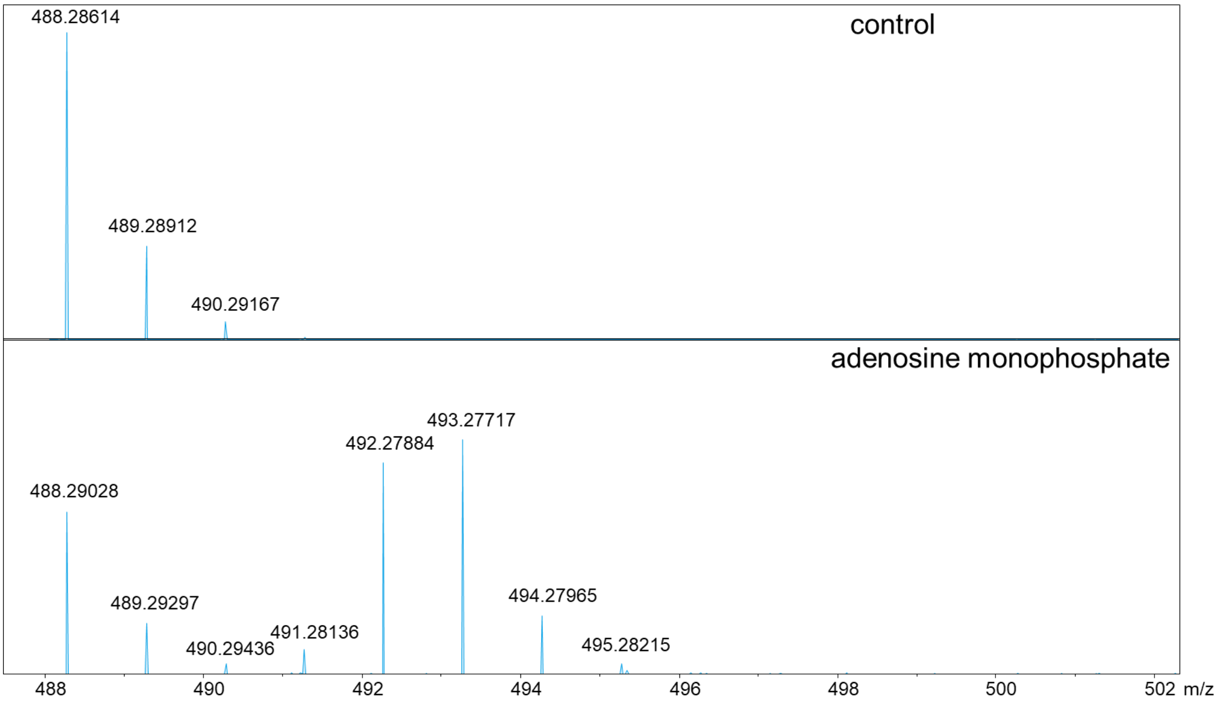

Figure S6. Partial ESI+MS spectra for 2-hydroxysorangiadenosine (**2**) supplemented with adenosine monophosphate ($^{15}N_5$) (bottom) and culture broth without precursor feeding as control (top).

1.2 Preparative LC-MS spectra

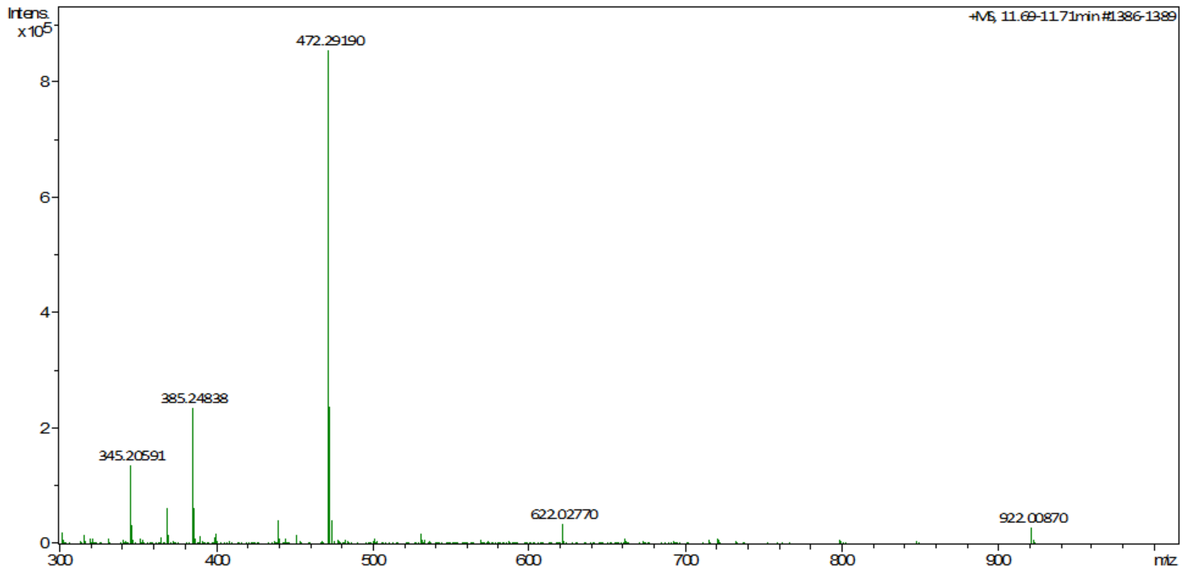

Figure S7. Partial ESI+MS spectra for sorangiadenosine (**1**).

molecules MDPI

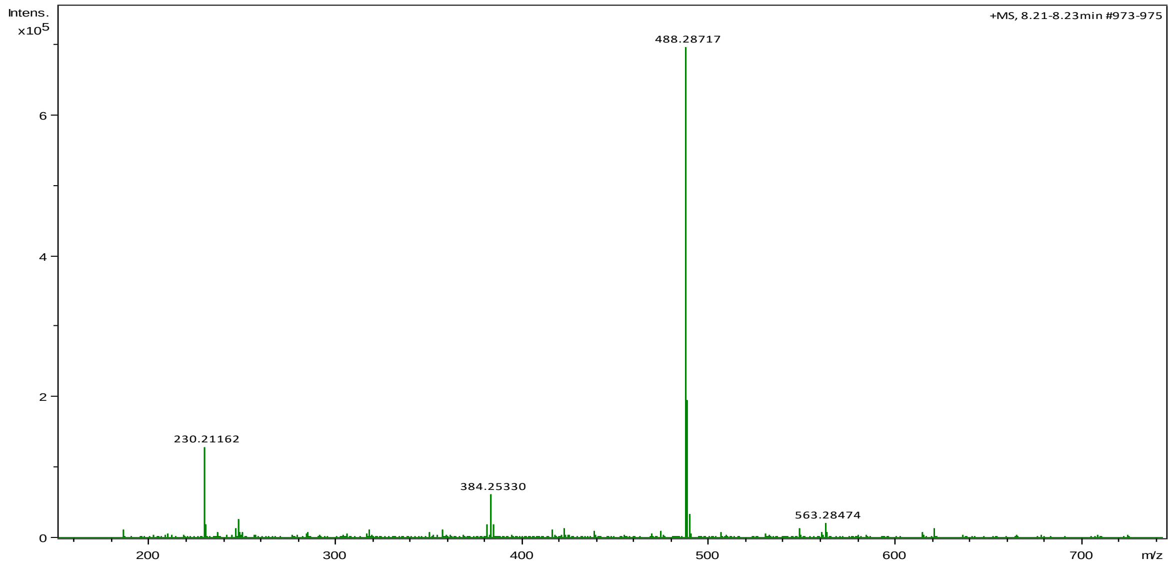

Figure S8. Partial ESI+MS spectra for 2-hydroxysorangiadenosine (**2**).

1.3 Alternative producer investigation

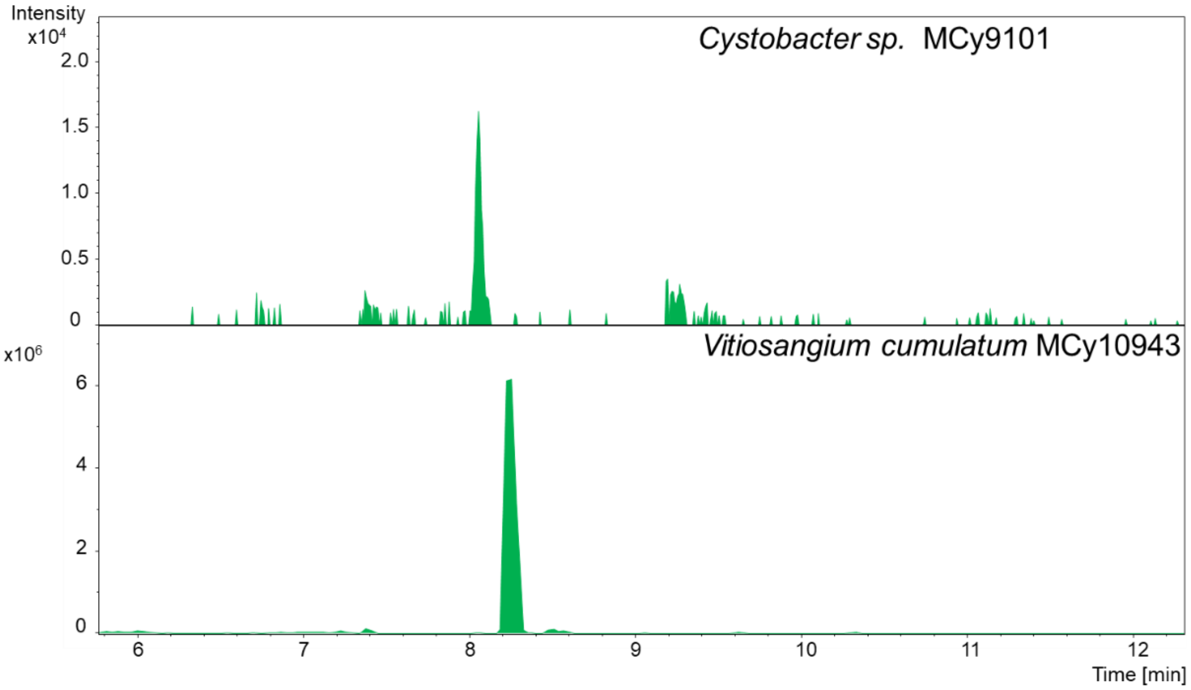

Figure S9. HPLC-MS EICs of crude extracts of *Cystobacter* sp. MCy9101 (top) and *Vitiosangium cumulatum* MCy10943^T (bottom). EIC: Extracted ion chromatogram, green: 488.2873 m/z, with a width of 7.9 ppm, 2-hydroxysorangiadenosine $[M+H]^+$.

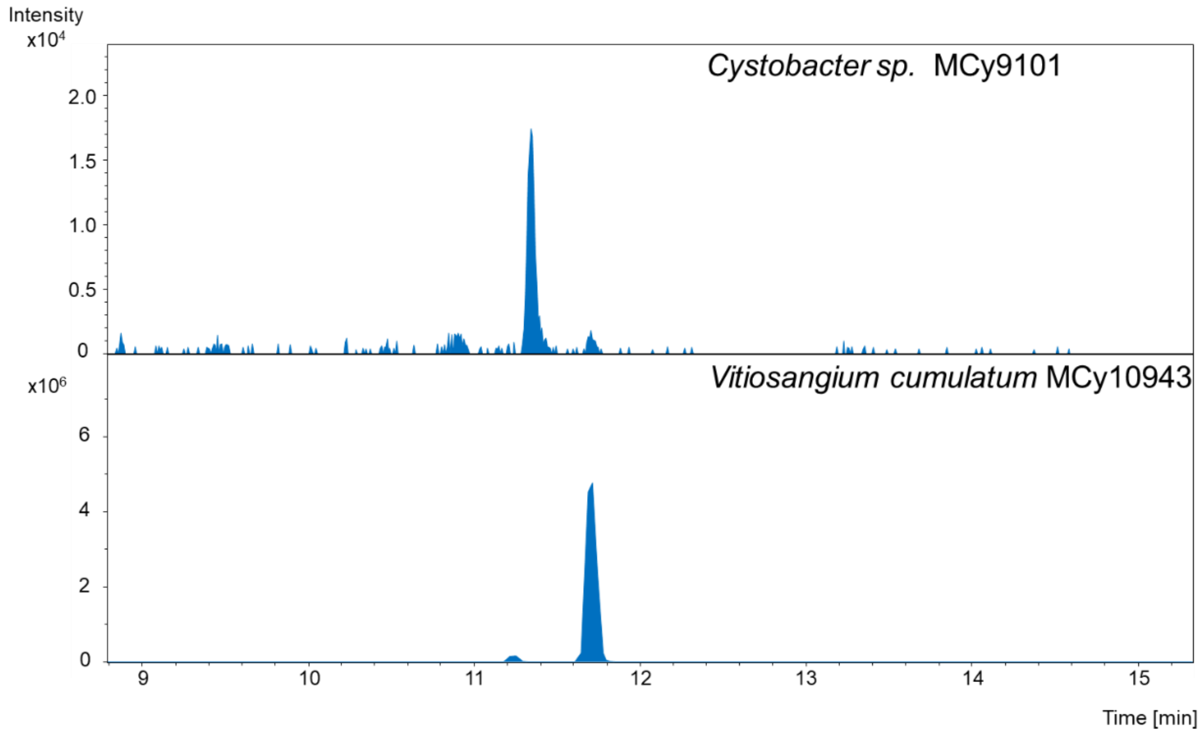

Figure S10. HPLC-MS EICs of crude extracts of *Cystobacter* sp. MCy9101 (top) and *Vitiosangium cumulatum* MCy10943^T (bottom). EIC: Extracted ion chromatogram, blue: 472.2924 m/z, with a width of 7.9 ppm, sorangiadenosine $[M+H]^+$.

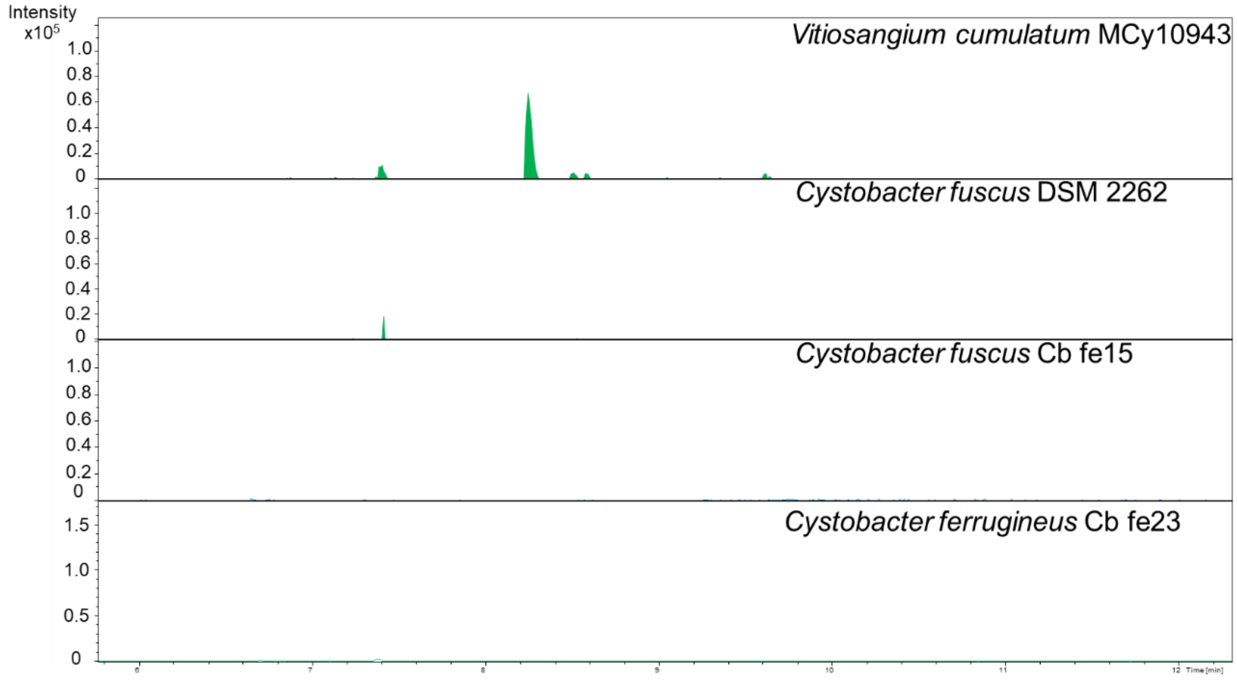

Figure S11. HPLC-MS EICs of crude extracts of *Vitiosangium cumulatum* MCy10943^T, *Cystobacter fuscus* DSM 2262, *Cystobacter fuscus* Cb fe15 and *Cystobacter ferrugineus* Cb fe23. EIC: Extracted ion chromatogram, green: 488.2873 m/z, with a width of 7.9 ppm, 2-hydroxysorangiadenosine $[M+H]^+$.

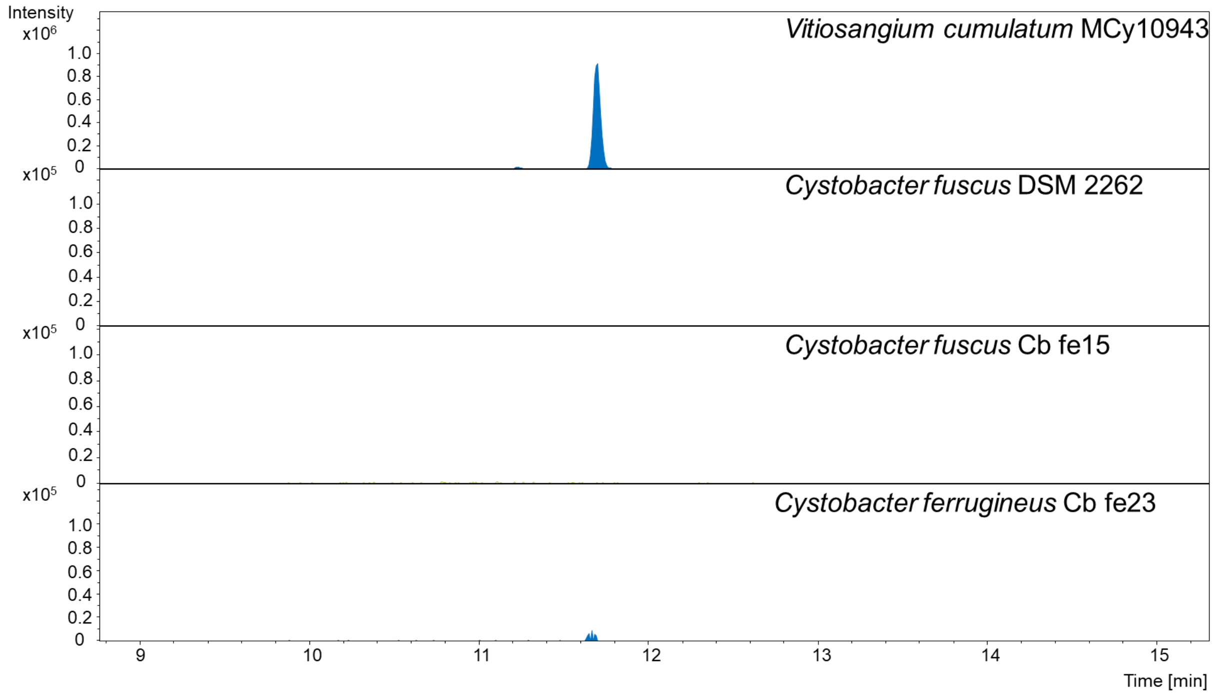

Figure S12. HPLC-MS EICs of crude extracts of *Vitiosangium cumulatum* MCy10943^T, *Cystobacter fuscus* DSM 2262, *Cystobacter fuscus* Cb fe15 and *Cystobacter ferrugineus* Cb fe23. EIC: Extracted ion chromatogram, green: 472.2924 m/z, with a width of 7.9 ppm, sorangiadenosine $[M+H]^+$.

molecules MDPI

1.4 CD spectrum

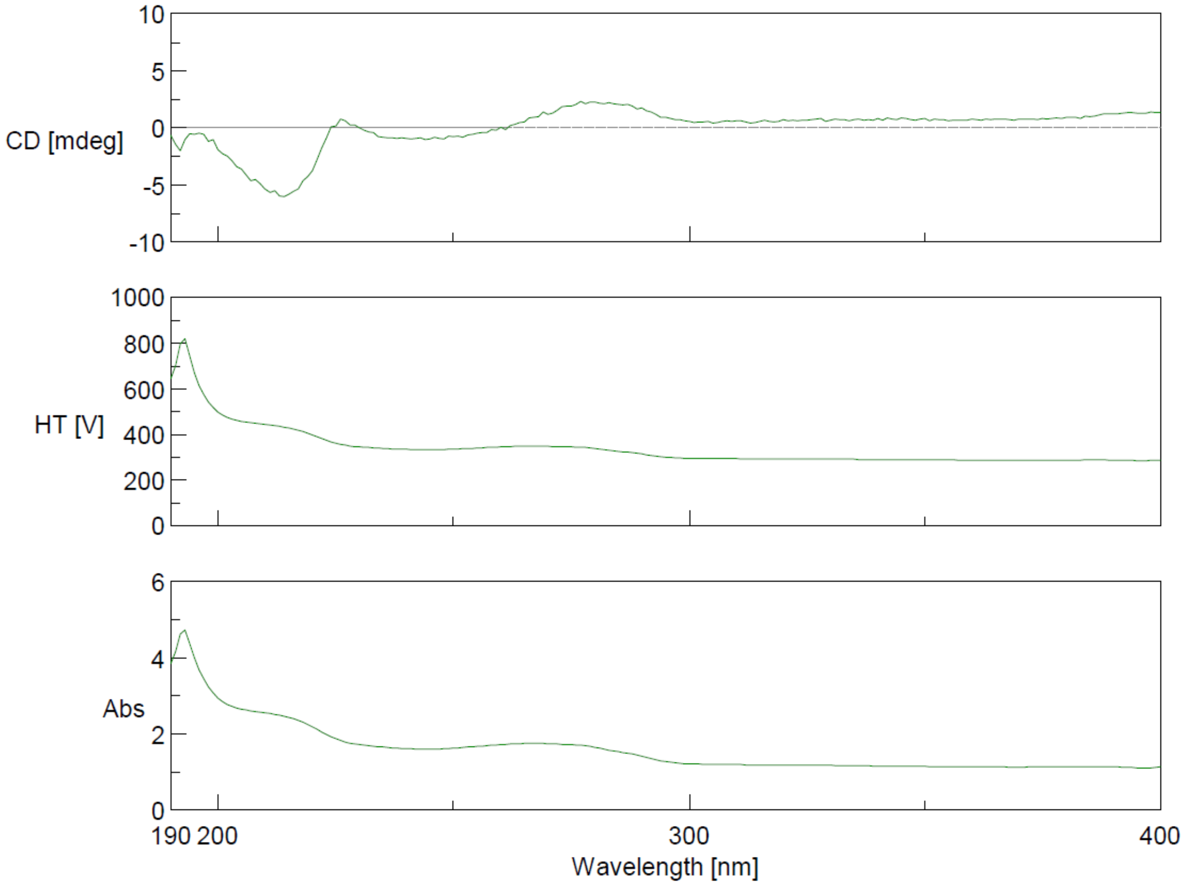

Figure S13. CD spectrum of 2-hydroxysorangiadenosine at 1.0 mM in MeOH in the area 190–400 nm, shows two negative cotton effect bands at 215 nm and 260 nm (top).

2. Biosynthetic *in silico* investigation

2.1 Leucine degradation (and alternative isovaleryl CoA biosynthesis)

In myxobacteria, the essential terpene and terpenoid precursors isopentenyl (pyro)/diphosphate (IPP) and dimethylallyl diphosphate (DMAPP) can be biosynthesized through the mevalonate pathway, but also through degradation of leucine. This leucine degradation pathway has been identified and characterized previously in the myxobacteria *Myxococcus xanthus* [1,2] and *Stigmatella aurantiaca* Sg a15 and DW 4/3–1 [3] (**Fig. S14**).

The genes in the myxobacterium *M. xanthus* DK1622, which encode the proteins involved in leucine degradation and the alternative biosynthesis of isovaleryl-CoA [2] have been described previously. For that reason, the genome sequence of *Vitiosangium cumulatum* MCy10943^{T} was investigated accordingly to find homologues genes. All genes encoding proteins which are required for the leucine degradation pathway and the alternative biosynthesis of isovaleryl CoA from *M. xanthus* DK1622 and their corresponding homologues in *Vitiosangium cumulatum* MCy10943^{T} are listed in **Table S1**.

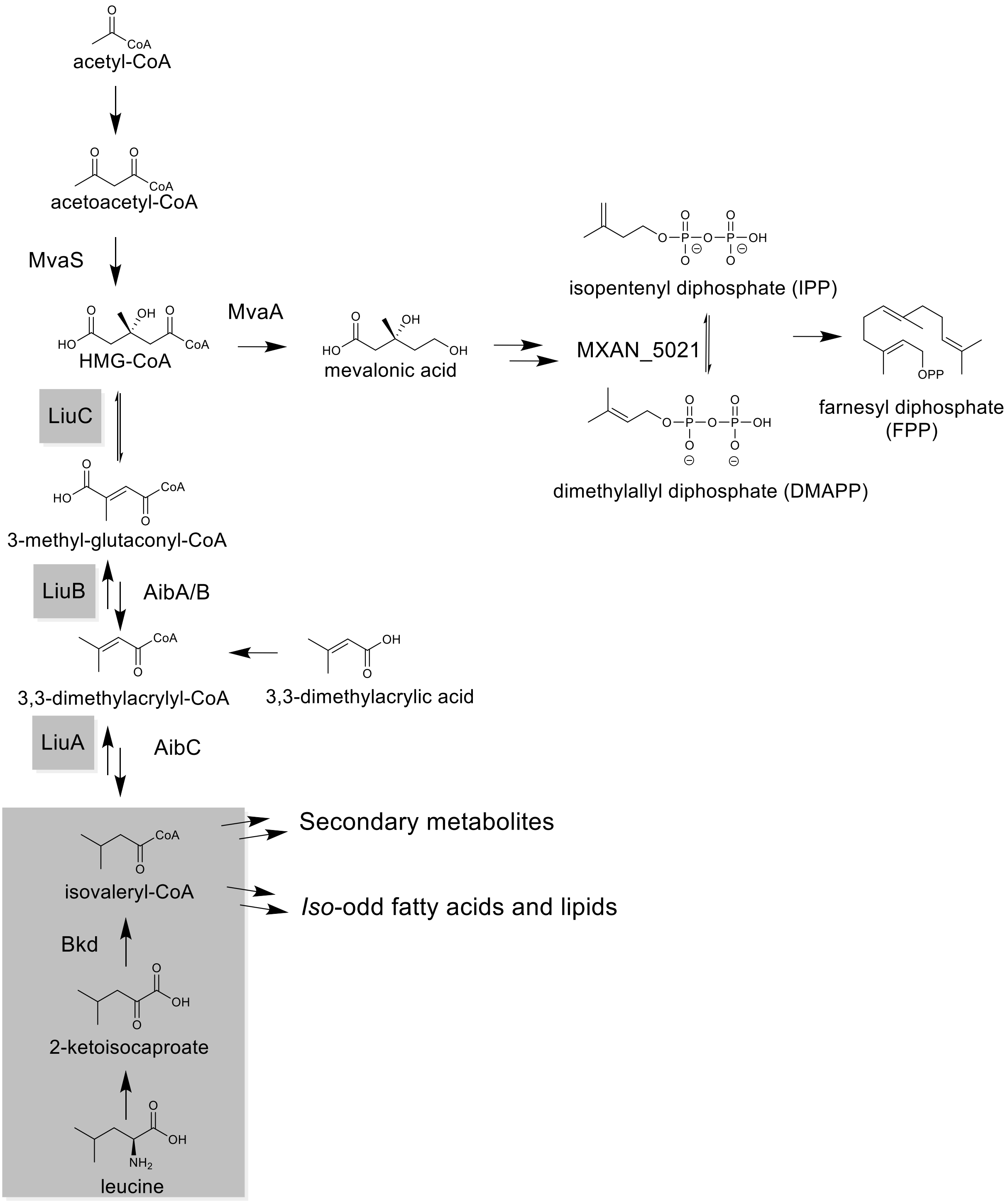

Figure S14. Leucine degradation pathway (grey boxes) and the alternative biosynthesis of isovaleryl CoA observed in *Myxococcus xanthus* DK1622. Proteins are listed in **Table S1**.

Table S1. Proteins involved in leucine degradation and in the alternative isovaleryl-CoA biosynthesis in *Myxococcus xanthus* DK1622 and their identified homologues in *Vitiosangium cumulatum* MCy10943[T].

Protein	Gene locus	Size (aa)	Deduced function	Coverage/ Similarity (%)
LiuA	MXAN_3760	381	short chain acyl-CoA DH	100/88.2
LiuB	MXAN_3759	513	carboxyl transferase (α & β SU)	99.81/90.1
LiuC	MXAN_3757	258	3-methylglutaconyl-CoA hydratase	100/85.7
AibR	MXAN_4263	228	TetR-like transcriptional regulator	82.89/76.2
AibA[a]	MXAN_4264	265	MG-CoA decarboxylase	100/80.8
AibB[a]	MXAN_4265	246	MG-CoA decarboxylase	100/84.6
AibC	MXAN_4266	345	dehydrogenase, Zn binding	99.71/84.0
MvaS	MXAN_4267	418	HMG-CoA synthase	99.76/82.3
BkdE1a	MXAN_4564	336	2-oxoisovalerate DH complex E1 α SU	100/80.4
BkdE1b	MXAN_4565	352	2-oxoisovalerate DH complex E1 β SU	100/84.9
MvaA	MXAN_5020	442	HMG-CoA synthase	99.55/81.1
–	MXAN_5021	352	isopentenyldiphosphate isomerase	99.72/82.9

[1]Liu [1]: leucine and isovalerate utilization// Aib [2]: alternative isovaleryl-CoA biosynthesis/ AibA/AibB: Catalytically active as complex [4]. Bkd: (branched chain keto acid dehydrogenase).

Amino acid sequence of enzymes involved in leucine degradation and alternative isovaleryl-CoA biosynthesis homologues in MCy10943[T]

>**LiuA homologue:**
MDFELPESHRALQSSLRDFCERKVKPYAREWDKEEKFPMEVVRELGELGVLGMLVSEEYGGAAMDSL
AVAVAVEEIARYDGSLALTVASHNGLGTSHLRVFGNDAQRRKYLPKLATGEYLGAWGLTEPGSGSDA
AGMKTTAVRKGDKWVLNGTKMFITQGTVGSVFVVLAVTSPEKKQKGITAFILEKGLPGFSQRSIHGKL
GMRSSDTAELVMENVEVPDSQRLGEVDHGFIDTMKILDKGRITIGALAVGLARGALEESVRYAQERTA
FGQPIADFQGLRWMFADMKTETDAARLLVHRAAFLADAGQPYSQEASMAKLFASEVATRACGKAVQ
IHGGYGYTREFPVERYLRDAKLCEIGEGTSEIQRTIIAREVFKKA

>**LiuB homologue:**
MPPGAMSFDQKLLEKIAQVEKGGAEKYHAKNRETGKLFARERIRLLVDEGSFVEDAKLANNLDAELPS
DGVIIGVGKVAGRAVAIMANDSTVKAGSWGARTVEKILRIQETAKQLRCPLMYLVDSAGARITDQVE
MFPGRRGAGRIFYNEVHMSGEVPQVCLLFGPSAAGGAYIPAFCDLVIMVDGNASMYLGSPRMAEMVI
GEKVTLEEMGGAKMHCSVSGVGDVLVKTEQEAIEAAKKYISFFPENFTQVPPRADPRAPKSSGKRVDE
IIPADQNKPFDMYALINELIDEGSWFEVKKLFAQELITGLARIDGRPVGIVANQPKYKGGVLFVDSADK
AARFIWLCDAFNIPLLYLADVPGFMIGTKVERAGIIRAGAKMISAVSEASVPKICVVVRKAYGAGLYAM
SGPGFAPDATLALPQAMIAVMGPEAAVNAVYFNKIQEKPEAERAAYVQQLRDEYRQDVDIYKLASEL
VVDEIVPGDRLRHELQQRYELYSRRFQPRETKKHGVYPV

>**LiuC homologue:**
MAEFKVDARGPIEIWTIDGEGRRNAISRAMLKEFEEMVARVSHGHDTRAVVVTGAGDKAFCAGADLK
ERSTMSEPEVRAFLDGLRRTLRSIEKSDCVFIAAINGAAFGGGTELALSCDLRVAAPAAELGLTEVKLGI
IPGGGGTQRLTRLVGPGRAKDLILTGRRLNAAEAFSIGLVNRLAPEGHLLDTAYSMAESIVENAPIAVAT
AKHAIDEGLSLELDEALALELRHYEKVLATEDRLEGLKAFAEKRKPVYKGR

>**AibR homologue:**
MQSGRRPDDGERYRAILETAARLICERGFEGTSMQEIAAACRMTKAGLYHHVQNKEQLLFDIMSYGM
DAFEQQVLDKVRSNPDPVERLRECMRLNIHLVTGGCIKEVIIILHEHATLRGEARAFIDSRKKAYVRFLE

DSFSEAVRMGRIRPVQPTVAAFSFLGMVLWIYKWFQPDGRLSADQVANEMVELLFAGLVTPAAAAAP
GAGAPMLALVPPKAVGGES

>**AibA homologue:**
MRPARWGSVTELVASIPDGALLATGGFMLGRAPMALVLELIAQGRRNLQLISLPNPLPAEFLVAGGCL
ARVELPFGALNLEGRVRPMPCLKRAIEQGRIDWREHDGYRVVQRLRAASMGLPFIPAPDADVSELADA
EPLQTVVDPFTGQRVPVEPAVYPDVALIHARAADERGNLFIEDPTTDLLVAGAARRVLATAEERVTRLP
RVTIPGFQVERVALARDGALPTGCTGLYPHDDEMLADYLALAEAGREAEFLDSLLRTRRAA

>**AibB homologue:**
MSVESSVSPAEVVVSLLAREIDDGAVVATGVASPLAILAIAVARATHAPGLTYLACVGSLDPDLPCLLP
SSEDLGYLEGRSAEISIPDLFDHARRGRVDTVFFGAAEVDVQGRTNMTASGSLEKPRTKFPGVAGAATL
RQWVRRPVLVVPRQSRRNLVPEVQVVTTRDDRRPVKLISDLGVFELGAGGARLLSRHPWASLDTIGER
TGFSFQVEESLPVTPLPDARTVSAIRAIDSHRLRDQLVGA

>**AibC homologue:**
MKAVVLREFGEASNLRMESVPDPRPGRGEVLIRVHACGVCYHDVINRRGNLPRTHVPAILGHEAAGEV
VEVGPDTPGWKVGDRVATLQRLSCGECALCKSGRNSLCKKDNRFFGEEISGGYAQYMTAPVAGLGRV
PANLPWPVAATVCCTLGTAVHTVRTRARVRAGETVLITGASGGVGLAAVQLAKLDGARVIAVTSGEA
KVQPLREAGADEVIVSRGLDFAAETRKRTGGEGVNVAIEIVGSATFGQTLKAMAPGGRVVVVGNLESG
IVELNPGLVIVKELEILGAYATTREELDESLRLTADGKIRPFVSESVPLAEAARAHFRLENREIAGRLVLV
PPELQ

>**MvaS homologue:**
MKKQVGIEALAIAVPRRYVDIEDLARARGVDPAKFTVGLGAKEMAVADPGEDSVALAATAAARLIQR
NDVDPAKVGMLVVGTETGVDHSKAVASHVQGLLKLPRSMRTYDAQHACYGGTAGLMAAVEWIASG
AGAGRSAIVVCSDIARYGLNTAGEPTQGGGAVALLVSEQPDLLALDIGLNGVCSNDVYDFWRPLGRRE
AVVDGHYSISCYLEALSGAYRGWRERALAHEVVRWGETLPGEQLARILYHVPFCKMARKAHTQLRLC
DLEDAPGAGASTPAAREEIAKSSASYDAQVASSLGINARVGNVYTASLYLALAGLLQGESAALAGKRI
GLLSYGSGCCAEFYSGVVGAAAAQRMAKADVESVLAKRERITLEEYERIMRLSSDAPERVNPASGEFR
LVEIRDHRRIYAAG

>**BkdE1a homologue:**
MPKPRLLNREEDSLPLERELLVRMHDLMVKARVLEERLIQMYKQGHGYFWIGGPGEEAFNVPLGLLM
KKGQGPAYDYLHAHYRQSATLLALGEEPIGSLRQMKNTATDPYSGGRNFAGHYSKREWNIAPVSSPIE
VQYVMAPGTALAQKRHGGDGITIVTGGDAGTAEGDFASCLIWSSRPGNELPLLIIVTNNKWGISTPAET
QHGETHVADRGKAFNIRSKTIDGNDPINAYLELKEAMEYVRKERKPFLMEAMVSRLYGHSSASGANF
VGNELDCLARFEERLEKNGVLTRKEMDDLRNRYTEDMAAMARQVREEPLPAPETIWNHIFAERK

> **BkdE1b homologue:**
MANMAQAIRMALHYAEENLGVTDVFGEDVGAPLGGVFTATQGLKTAWNSPLDERGIIGMAIGLAMA
GQKPVAEIQFADYIYNTIDLLKIAGNTCWSTHGDWNVPMVVRTPVGSGIRGSIYHSHSFDATATHIPGW
KIVMPSNPLDAYGLLISACQEINPVMYLEPKALLRIKGEERIPGEPDDDKLLSKMIDAPLGDRSQWKPQ
WPQQLEAYAVPIGKGKVVRSGSQVTVVSYGRTLPLCKKAADDLASAGVDAEVIDMRTLWPYDWELI
KGSVEKTGRVLYVNEDTEVTNFGEHLVRRTVEELFFKLVAPPRLLAGKFVPGIGLADTLEMASVPQLG
DITAAIRSLAAEQP

molecules MDPI

>MvaA homologue:
MSETLTSRLSGFHKLSMVERHEKLAQMLGLDEMDLAQLQGISALQPGLANQMIENAVGTFSLPLGLGL
NLQINGRDYLVPMAVEEPSVVAAVSFASRIVREAGGFSAEADDPIMIGQVQLTRYGDPTEATKKILAAK
EALLALANSFHPSMVKRGGGCKDIEVRVLPAPEGPRGEPLLVVHLIIDTQEAMGANLINTMAEGVAPLL
EQLTGGKVFLRILSNLADRRLARATCRIPVEALADFDMPGHVIAEGIYQASRFALADPYRAATHNKGIM
NGIDSAAIAAGQDWRAIEAGAHAFACRDGQYRPLSTWHVDDGHLVGRIELPMALGLVGGPIKVHPGV
QVAMKILRVESARELAMVFAAVGLAQNFAAVRALGSIGIQKGHMALHARCVAVTAGARGDWVEKIA
ELLVSAGHVKVEKAREIIASLSAEDFRAATGTDS

>MXAN_5021 homologue:
MVPKQGRRDVTGEEATAKRKDAHLDLCATGEVEPAENSTLLEYVRLVHCAMPEMAVEEVDLSTEFLG
KKLRYPLLITGMTGGTERAGVVNRDLALLAERHGLAFGVGSQRAMAENPQAAETYVVRHVAPTVPLL
GNIGLYQAIELGVDGVRRLADAIGADGMALHLNAGQELTQPEGDRDFRGGYTVVEGLVRVFGARLLV
KETGCGIGPEVARRLVDLGVRNLDVSGLGGTSWVRVEQLRASGVQAQVGAEFSSWGIPTAAAIASVRR
AVGPEPRLVASGGLRTGLDAAKVLALGADLAGMALPLFRAQQAGGLEGAEQALAVILSGLRQALVLT
GSRNCGELRQKPRVIMGQLKDWLAAL

2.2 Formation of sesquiterpene scaffolds and eudesmane-type sesquiterpenes

The formation of the sesquiterpene scaffold starts from farnesyl pyrophosphate (FPP) with subsequent cyclization via a sesquiterpene cyclase, which is known to catalyze the formation of diverse (poly)cyclic sesquiterpene skeletons [5]. Six different initial cyclization reactions are possible starting from farnesyl diphosphate [5] (**Fig. S15A**). These include the direct conversion of farnesyl diphosphate to (*E*,*E*)-germacrenyl cation via 1-10-cyclization or 1-11 cyclization to the (*E*,*E*)-humulyl cation. The 2-*E* double bond of FFP can be isomerized via ionization leading to the farnesyl cation, followed by reattachment of diphosphate at C-3 to yield nerolidyl diphosphate (NPP) (**Fig. S15B**). This allows NPP a 1,6-cyclization to the bisabolyl cation, a 1,7-cyclization to the cycloheptenyl cation, a 1,10-cyclization to the (*E*,*Z*)-germacrenyl cation or a 1,11-cyclization to the (*E*,*Z*)-humulyl cation (**Fig. S15C**).

According to the featured eudesmane-type sesquiterpene structure, a logical proposal would start from FFP to yield via a 1,10-cyclization the (*E*,*E*) germacrenyl cation (**Fig. S16A**). The germacrenyl cation could also be cyclized to intermedeol, such as described in the literature for intermedeol cyclases (**Fig. S16B**) [6]. However, the secondary metabolome of MCy10943^T shows no signal corresponding to the sum formula or mass of intermedeol or a hydroxylated eudesmadiene intermediate. On the contrary, three compounds with the mass of 205 m/z and the sum formula $C_{15}H_{25}$ are present in the crude extract of MCy10943^T, which correspond to the non-hydroxylated eudesmadiene intermediates (**Fig. S16C**, **Fig. S17**). Cyclization of eudesmadiene begins with a 1-10 bond

formation to yield germacrenyl A which is then protonated at C_6 to facilitate C_2-C_7 bond formation resulting in the eudesmane carbocations.

During the process, the C_2-C_3 bond of the farnesyl diphosphate retains its *trans* configuration. Elimination of protons at α, β or γ to the (*S*)-eudesmyl carbocation center accounts for the formation of α-selinene, β-selinene and selina-4(11)-diene. Elimination of protons at α, β or γ to the (*R*)-eudesmyl carbocation center accounts for the formation of alternative products like 8a-*epi*-α-selinene, selina-4(11)-diene. Considering the stereochemistry of the products it is assumed that **1** derives from the (*S*)-eudesmyl carbocation in the second stage.

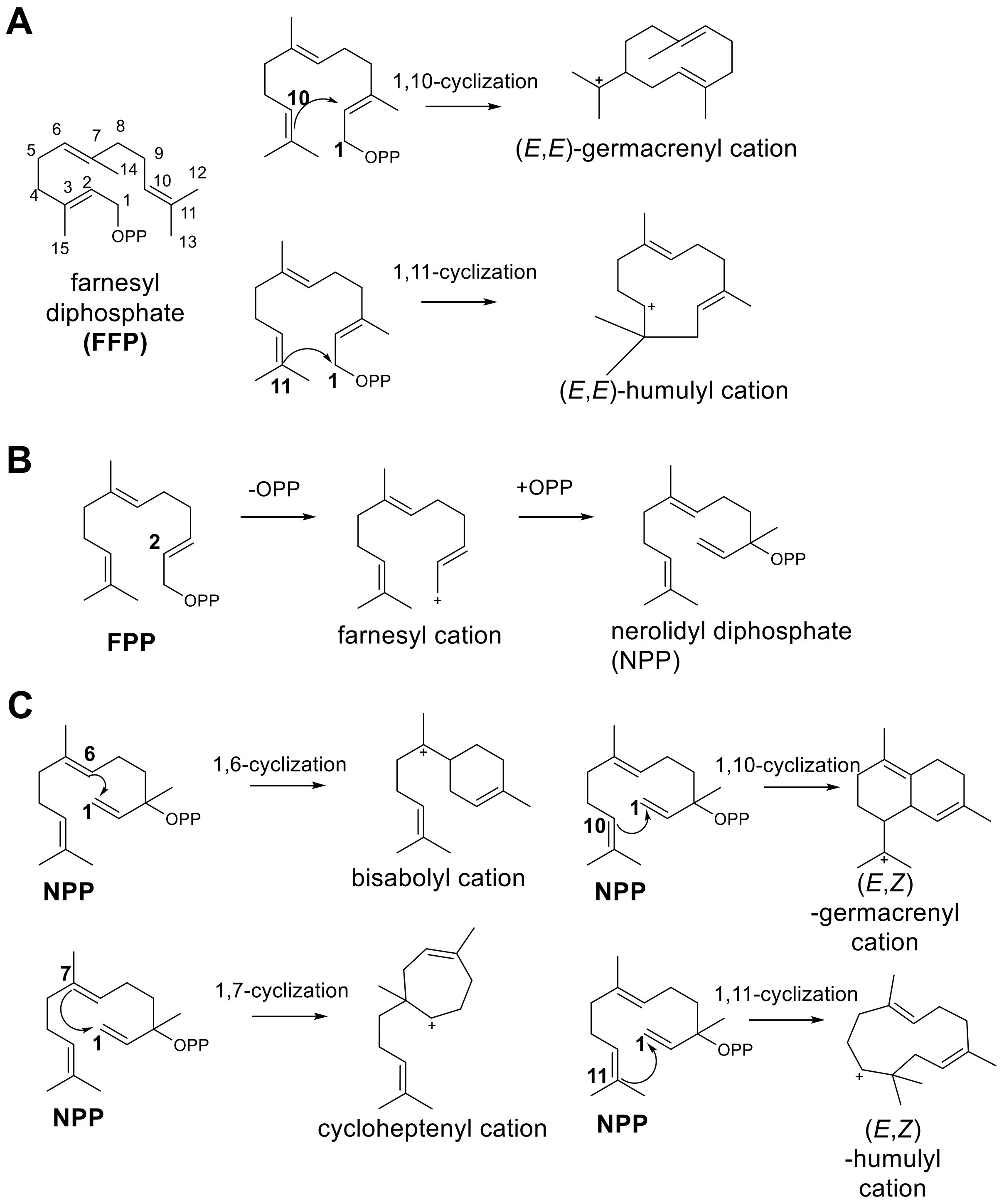

Figure S15. 1,10-cyclization and 1,11-cyclization from farnesylpyrophosphate (FFP) yield the (*E*,*E*)-germacrenyl cation, which is the proposed precursor for the generation of eudesmane-type sesquiterpene required for the biosynthesis of **1** and **2** or (*E*,*E*)-humulyl cation, respectively. Both enantiomers of the (*E*,*E*)-germacrenyl cation can be formed. **B**) Isomerization mechanism of FFP to nerolidyl diphosphate (NPP). **C**) NPP can be cyclized to a bisabolyl cation via 1,6-cyclization, to the cycloheptenyl cation via 1,7-cyclization, to the (*E*,*Z*)-germacrenyl cation via a 1,10-cyclization or to the (*E*,*Z*)-humulyl cation via a 1,11-cyclization. Both enantiomeric products of the 1,6-cyclization, 1,10-cyclization and 1,11-cyclization can be generated.

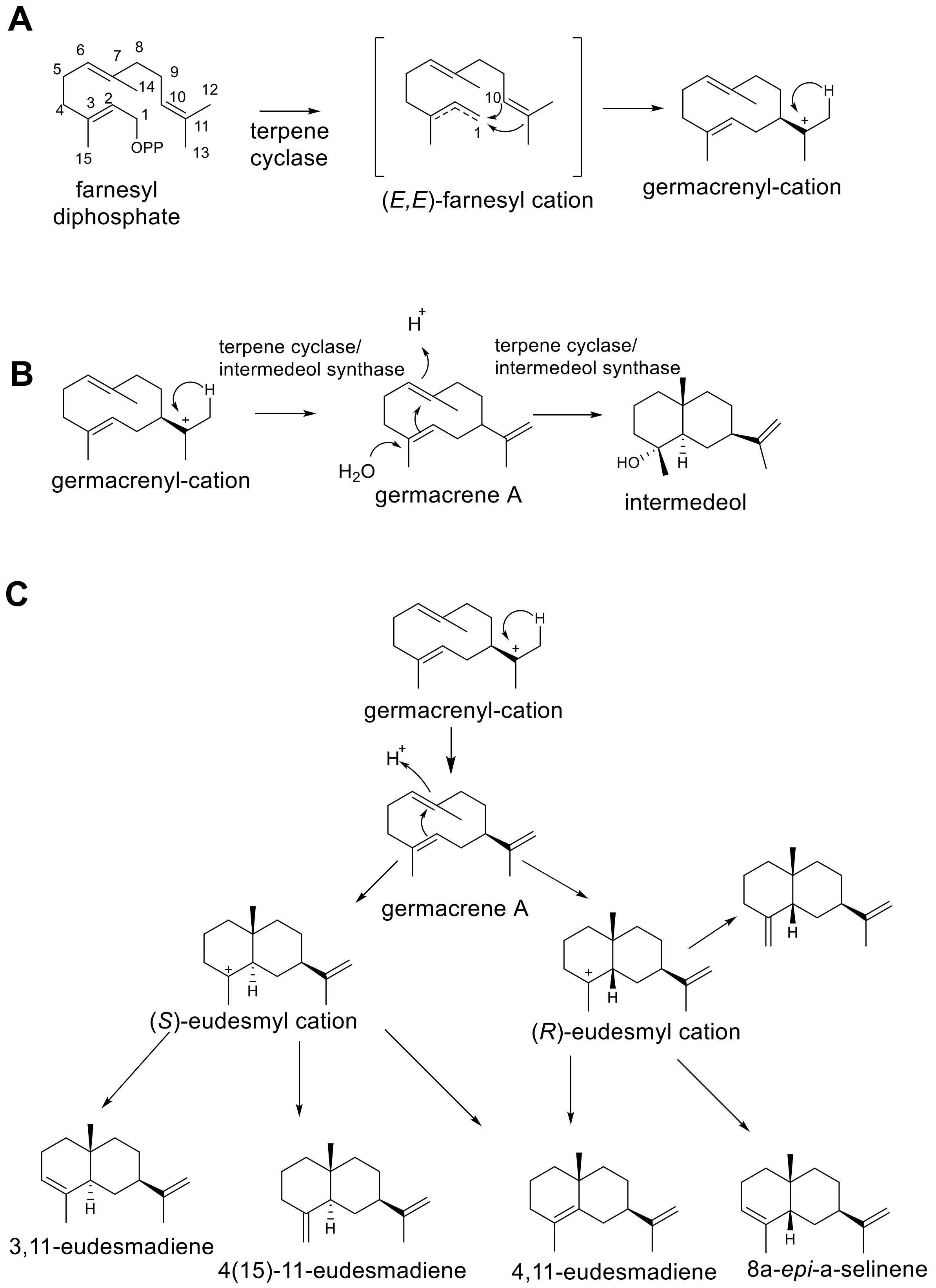

Figure S16. A) Proposed start of the sesquiterpene part during the biosynthesis of sorangiadenosine yielding the intermediate germacrenyl-cation. **B**) Possible formation of intermedeol from the intermediate germacrenyl-cation. **C**) Proposed formation of the different eudesmadiene building blocks.

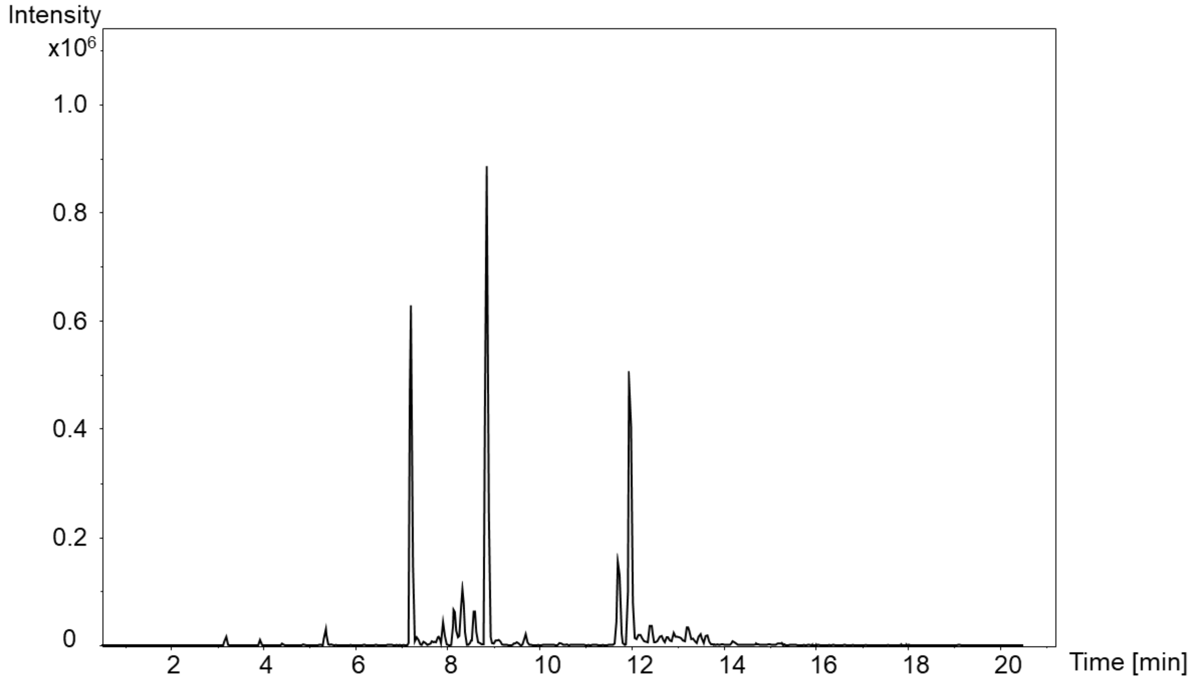

Figure S17. HPLC-MS EIC of *Vitiosangium cumulatum* MCy10943^T crude extract. EIC: Extracted ion chromatogram, black: 205.1956 m/z, with a width of 7.9 ppm, proposed non-hydroxylated eudesmadiene intermediates $[M+H]^+$.

2.3 Metabolome-genome correlation

Initial *in silico* analysis already excluded two of the antiSMASH-identified biosynthetic gene clusters (BGCs) to produce **1** and **2**, since both terpene cyclases share high sequence similarity to the geosmin terpene cyclase. In addition, a study investigating different terpene cyclases from *S. cellulosum* So ce56 suggested, that the geosmin cyclase gene (*sce1440*) is specific in terms of its production profile [7]. In order to narrow down the number of suitable BGCs for the production of **1** and **2** we performed metabolomic profiling of the myxobacterial strains *Cystobacter ferrugineus* Cb fe23, *Cystobacter fuscus* Cb fe15 (DSM 52655) and *Cystobacter fuscus* DSM 2262^T. These three strains harbor according antiSMASH and manual evaluation similar terpene gene clusters to No. 3, 5 and 6 (**Tab. 1**). In the respective crude extracts, no metabolites corresponding to the retention time, exact molecular mass and sum formula connected to **1** and **2** was found in the LC-MS chromatogram of these three myxobacterial strains (**Fig. S11** and **S12**). In addition, the gene clusters No. 3, 5, 6 were further excluded, since as proposed above, each gene cluster is missing at least either a gene encoding an oligoprenyl transferase or a cytochrome P450 gene. Taken together, genomic investigation combined with metabolomic profiling seemingly reduced the likelihood that the terpene gene clusters No. 3, 5 and 6 are responsible for the production of **1** and **2.**

molecules MDPI

However, since genes responsible for the production of the terpenes might be poorly expressed in some organisms and genes pivotal for the formation of the terpene scaffold can be located outside of the biosynthetic core region (terpene or diterpene cyclase genes), it cannot be entirely excluded that the terpene gene clusters No. 3, 5 and 6 are in some way involved in the biosynthesis of **1** and **2**.

Consequently, this metabolome-genome correlation indicates that only the terpene gene clusters 1, 4 and 8 might be involved in the biosynthesis of **1** and **2.** The terpene cyclase in cluster No. 4 identified as pentalene cyclase could account for the biosynthesis of an eudesmane-type sesquiterpene according to structural relationship to known eudesmane/selinadiene forming terpene cyclases. In addition, at least one aldo-keto oxidoreductase within terpene gene cluster No. 4 could be responsible for the conversion of **1** to **2**, albeit this enzyme would rather change the redox state of the alcohol/ketone than install a hydroxyl group. However, the identified terpene gene cluster No. 4 lacks presumably a gene which might account for transferring the adenosine to the sesquiterpene. Terpene gene cluster No. 8 harbors neither genes responsible for hydroxylation of **1** leading to **2**, nor it features genes accounting for the transfer of adenosine onto the eudesmane-type sesquiterpene.

The alternative producers of **1** and **2** with available in-house genome sequences *Archangium* sp. strain MCy8383 (Ar3548) (GenBank Accession number MT520809), *Archangium gephyra* strain MCy8375 (Ar8082) (GenBank Accession number MT520808) and *Cystobacter sp.* strain MCy9101 (SBCb004) (GenBank Accession number MT520810) harbor a terpene BGC resembling the candidate 2-hydroxysorangiadenosine BGC. The proposed hydroxsorangiadenosine BGCs in *Archangium* sp. strain MCy8383 (Ar3548) and *Archangium gephyra* strain MCy8375 (Ar8082) are highly similar to the candidate gene cluster in *Vitiosangium cumulatum* MCy10943^{T}, whereas the homologue BGC in *Cystobacter sp.* strain MCy9101 (SBCb004) deviates significantly from the herein described gene cluster organization; nevertheless all three homologues BGCs are missing the gene *sora12*. The genetic organization of the 2-hydroxysorangiadenosine BGC in *Cystobacter sp.* strain MCy9101 (SBCb004) is reduced to *sora7–9*, *sora11–12*, *sora18–19* and *sora21–22*. **Figure S18** shows this truncated 2-hydroxysorangiadenosine BGC in *Cystobacter sp.* strain MCy9101 (SBCb004) and **Figure S9** and **S10** demonstrate that this truncated BGC is presumably sufficient to produce **1** and **2** in low concentration.

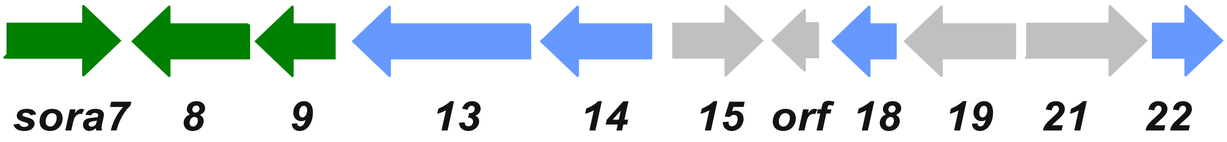

Figure S18. Genetic organization of the truncated 2-hydroxysorangiadenosine BGC from *Cystobacter sp.* strain MCy9101 (SBCb004).

molecules MDPI

The nucleotide sequence of the other eight putative terpene gene clusters from MCy10943^{T} have been deposited in GenBank under the following accession numbers:

- No2_MCy10943_terpene_gene_cluster (MT520812)

- No3_MCy10943_terpene_gene_cluster (MT520813)

- No4_MCy10943_terpene_gene_cluster (MT520814)

- No5_MCy10943_terpene_gene_cluster (MT520815)

- No6_MCy10943_terpene_gene_cluster (MT520816)

- No7_MCy10943_terpene_typeIIIPKS_gene_cluster (MT520817)

- No8_MCy10943_terpene_TfuA-related (MT520818)

- No9_MCy10943_terpene_RiPP_gene_cluster (MT520819)

3. Structure elucidation

3.1 NMR spectroscopic data

Table S2. NMR spectroscopic data for sorangiadenosine (**1**) in methanol-d_4.

		sorangiadenosine (**1**)			
**position**	δ_C, type	δ_H, (*J* in Hz)	**COSY-DQF**	**gCOSY**	**HMBC**
1	41.9	1.47 m<br>1.30 m	1.75	1.04, 1.59	2, 3,5,9,10,14
2	20.3	1.75 m<br>1.59 m	-	1.47, 1.30, 2.11	1, 3, 4
3	38.13	2.48 td (4.4/13.5)<br>2.11 br d (13.5)	1.75, 1.59	1.75, 1.30, 1.47	1, 2, 4, 5, 15
4	59.9	-	-	-	-
5	49.5	2.54 *dd* (2.0/12.5)	1.38	1.69, 1.38	3, 4, 6, 7, 10, 14, 15
6	27.9	1.69 m<br>1.38 d (12.5)	-	2.54, 1.94	5, 7, 8, 10, 11
7	47.7	1.94 m	1.69, 1.38	1.69, 1.38, 1.57	6, 8, 11, 12,13
8	28.3	1.57 m<br>1.49 m	-	1.94, 1.33, 1.47	6,7, 8, 9,10
9	46.5	1.47 m<br>1.33 m	1.49, 1.57	1.49, 1.57, 1.04	5, 8, 10, 14
10	35.8	-	-	-	-
11	151.7	-	-	-	-
12	21.1	1.68 s 3H	-	4.65	7, 11, 13
13	108.8	4.65 d 2H (16.1)	1.68	1.68	1, 7, 12
14	19.6	1.04 s 3H	-	1.30	1, 5, 9, 10
15	21.3	1.46 s 3H	-	1.75, 2.11, 2.48, 2.54	3, 4, 5, 6’
1’	-	-	-	-	-
2’	152.9	8.22 s	-	-	4’, 5’, 6’
3’	-	-	-	-	-
4’	148.7	-	-	-	-
5’	121.6	-	-	-	-
6’	155.9	-	-	-	-
7’	-	-	-	-	-
8’	141.1	8.24 s	-	5.94	1’’, 4’, 5’
9’	-	-	-	-	-
1’’	91.3	5.94 d (6.5)	4.74	3.74, 4.17, 4.31, 4.74	2’’,3’’, 4’, 8’
2’’	75.4	4.74 dd (5.4, 6.5)	4.31	4.31, 5.94	1’’, 4’’
3’’	72.7	4.31 dd (2.3/5.4)	4.17	4.17, 4.74	1’’, 4’’, 5’’
4’’	88.3	4.17 q (2.3)	3.88, 3.74	3.74, 3.88, 4.31	3’’
5’’	63.5	3.88 dd (2.3/12.6)<br>3.74 dd (2.3/12.6)	-	4.17	3’’, 4’’

Table S3. NMR spectroscopic data for 2-hydroxysorangiadenosine (**2**) methanol-d_4.

		2-hydroxysorangiadenosine (**2**)			
**position**	δ_C, type	δ_H, (*J* in Hz)	**COSY-DQF**	**gCOSY**	**HMBC**
1	50.8	1.80 dd (3.8/12.2)<br>1.30 t (9.1/12.2)	2.41, 3.94	1.06, 2.41, 3.94	2, 3, 5, 10, 14
2	65.6	3.94 m	1.27, 1.80, 2.52, 2.41	1.27, 1.80, 2.41, 2.52	1, 3
3	46.6	2.52 t (12.1)<br>2.41 m	3.94	1.46, 1.80, 3.94	2, 4, 5,15
4	60.4	-	-	-	-
5	48.5	2.60 *dd* (2.2/12.4)	1.70, 1.38	1.70, 1.38	3, 4, 6, 7, 10, 14, 15
6	27.6	1.70 m<br>1.38 t (12.4)	1.94, 2.60	2.60, 1.94	4, 7, 8,10, 11, 14
7	47.6	1.94 m	1.56, 1.48, 1.70, 1.38	1.70, 1.48, 1.56, 4.65	11, 12, 13
8	28.0	1.56 m<br>1.48 m	1.94, 1.52, 1.38	1.94, 1.38, 1.52	6, 7, 10, 11
9	46.4	1.52 m<br>1.38 t (12.4)	-	-	1,5, 8, 10, 14
10	35.6	-	-	-	-
11	151.5	-	-	-	-
12	21.1	1.68 s 3H	4.64	4.64	7, 11, 13
13	109.0	4.65 d 2H (15.4)	1.68	1.68	7, 11,12
14	20.7	1.06 s 3H	-	1.27, 1.38	1, 5, 9, 10
15	22.4	1.46 s 3H	-	1.94, 2.60	3, 4, 5, 6’
1’	-	-	-	-	-
2’	152.9	8.24 s	-	-	4’, 5’, 6’
3’	-	-	-	-	-
4’	148.8	-	-	-	-
5’	121.6	-	-	-	-
6’	155.7	-	-	-	-
7’	-	-	-	-	-
8’	141.2	8.26 s	-	5.94	1’’, 4’,5’, 6’
9’	-	-	-	-	-
1’’	91.3	5.94 d (6.5)	4.74	4.17, 4.74	2’’, 3’’, 4’’,4’, 8’
2’’	75.4	4.74 dd (5.1/6.4)	4.31	4.31, 5.94	1’’,3’’,4’’
3’’	72.7	4.31 dd (2.5/5.1)	4.17	4.17, 4.74	1’’, 4’’, 5’’
4’’	88.3	4.17 q (2.5)	3.88, 3.74, 4.31	3.74, 3.88, 4.31	3’’,5’’
5’’	63.5	3.88 dd (2.5/12.6)<br>3.74 dd (2.5/12.6)	4.17	4.17	3’’, 4’’

[1] Tables may have a footer.

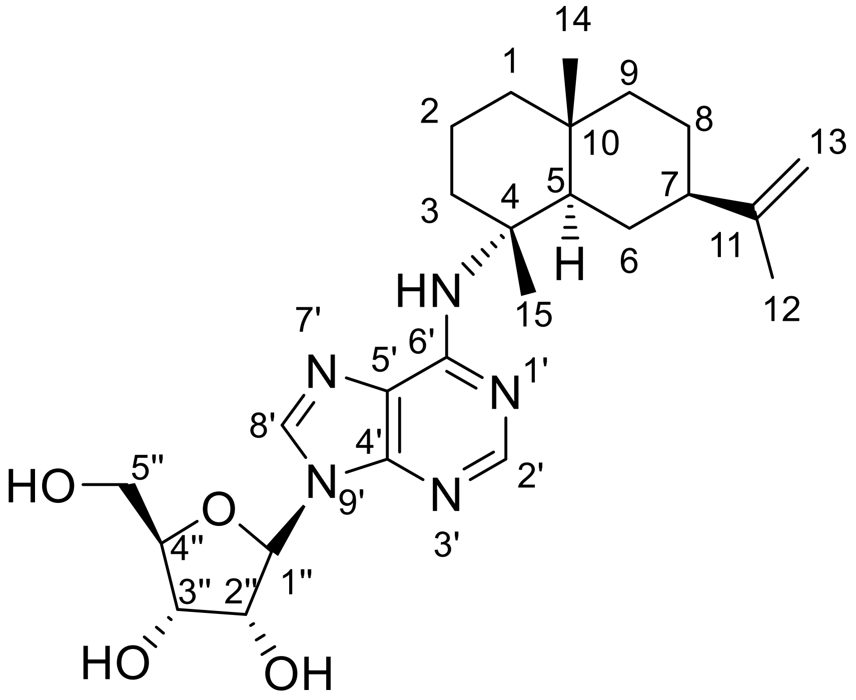

sorangiadenosine
1

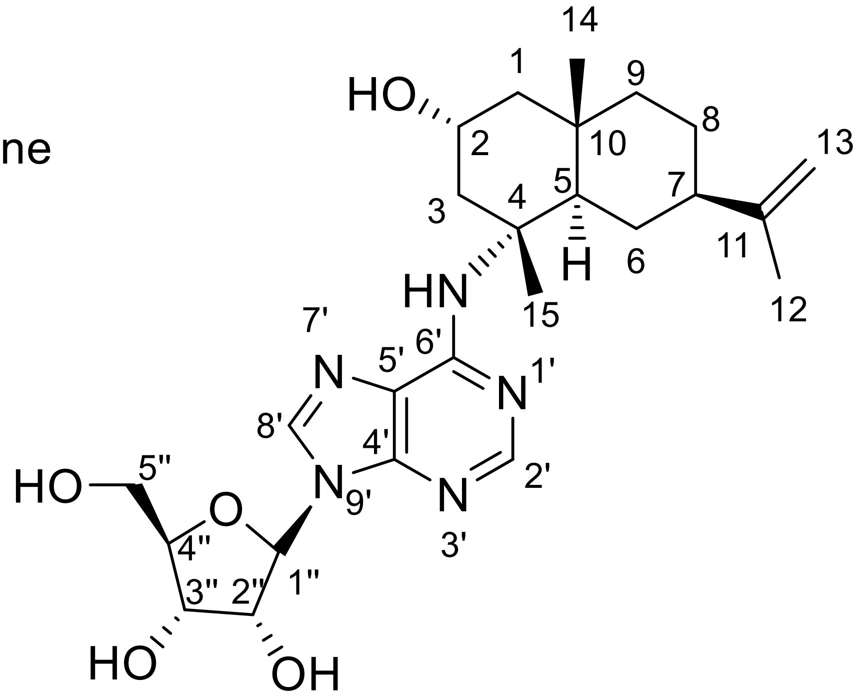

2-hydroxysorangiadenosine
2

Figure S19. Chemical structure and numbering of sorangiadenosine (**1**) and 2-hydroxysorangiadenosine (**2**).

3.2 ^{1}H and ^{13}C NMR spectra of sorangiadenosine and 2-hydroxysorangiadenosine

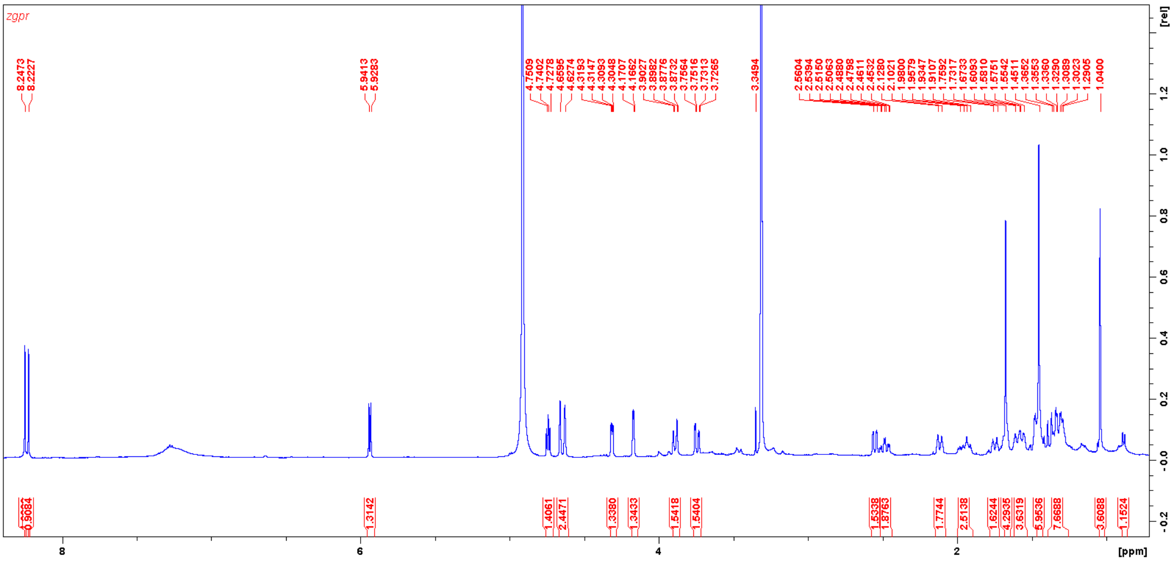

Figure S20. *^{1}H NMR* spectrum of sorangiadenosine (**1**) in methanol-d_4.

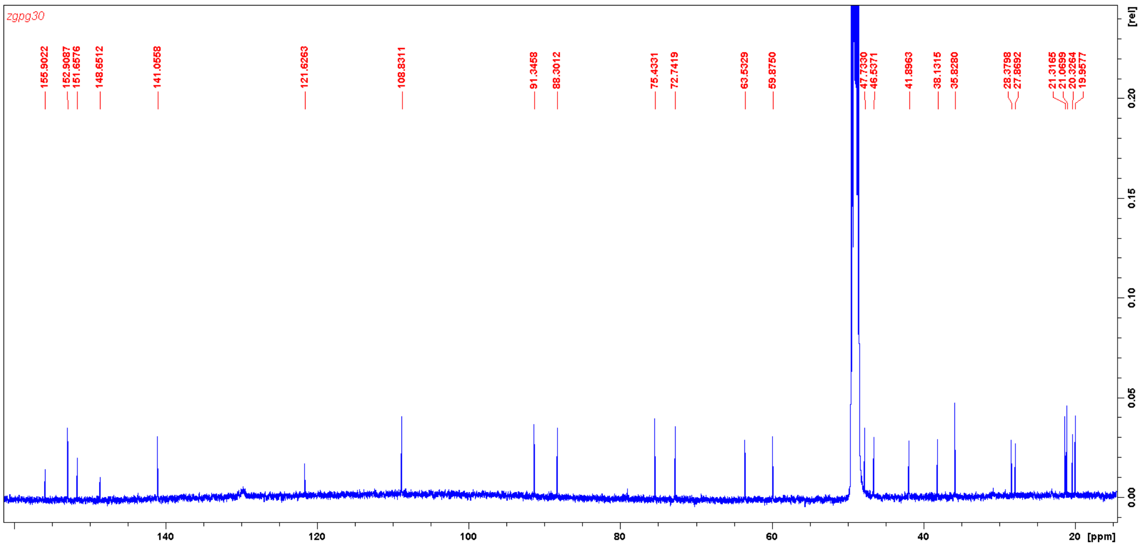

Figure S21. *^{13}C NMR* spectrum of sorangiadenosine (**1**) in methanol-d_4.

molecules

MDPI

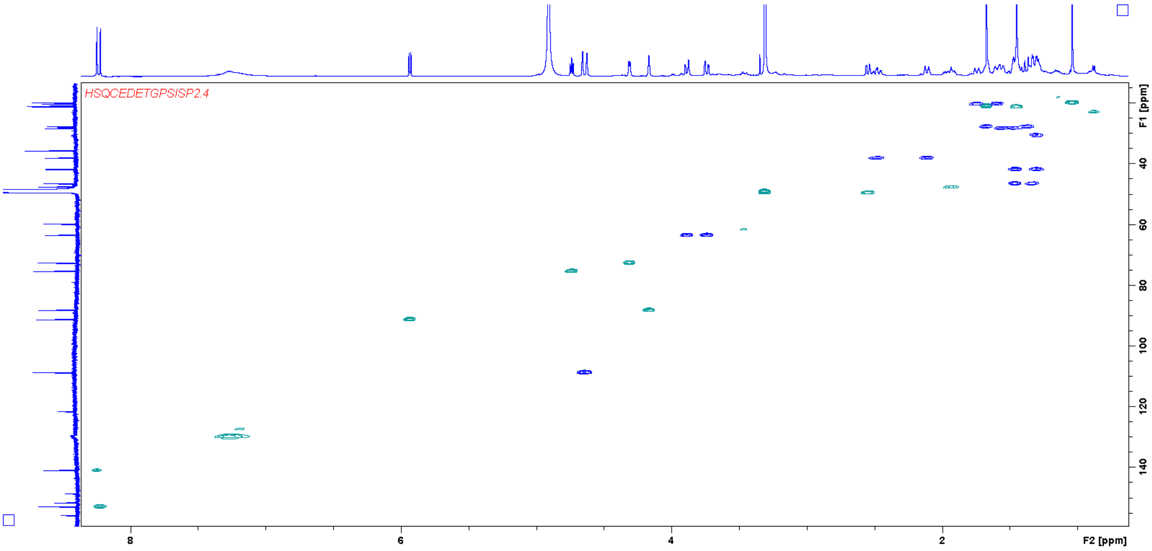

Figure S22. *HSQC* spectrum of sorangiadenosine (**1**) in methanol-d$_4$.

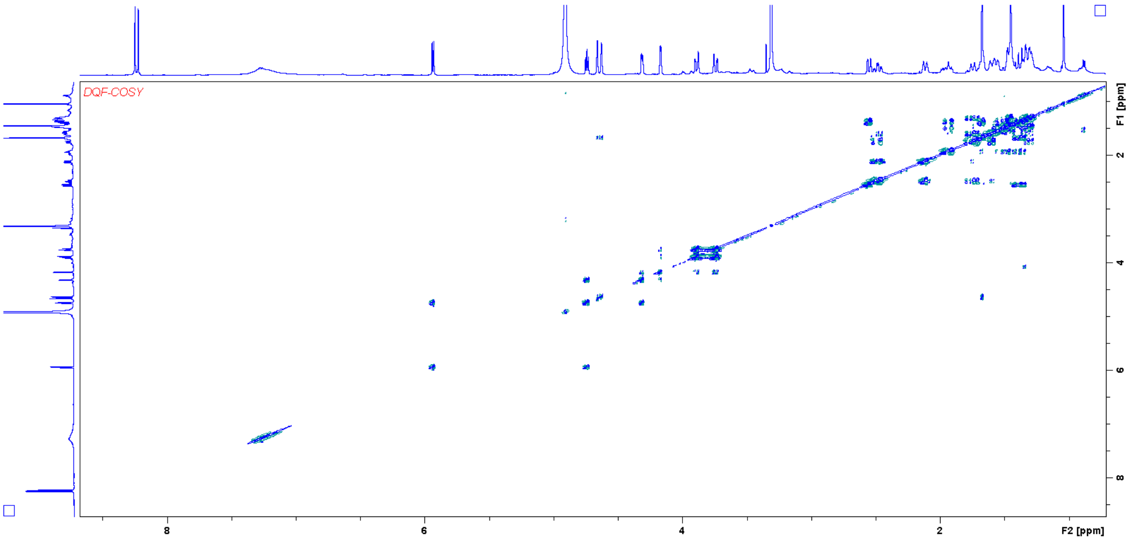

Figure S23. *DQF-COSY* spectrum of sorangiadenosine (**1**) in methanol-d$_4$.

molecules
MDPI

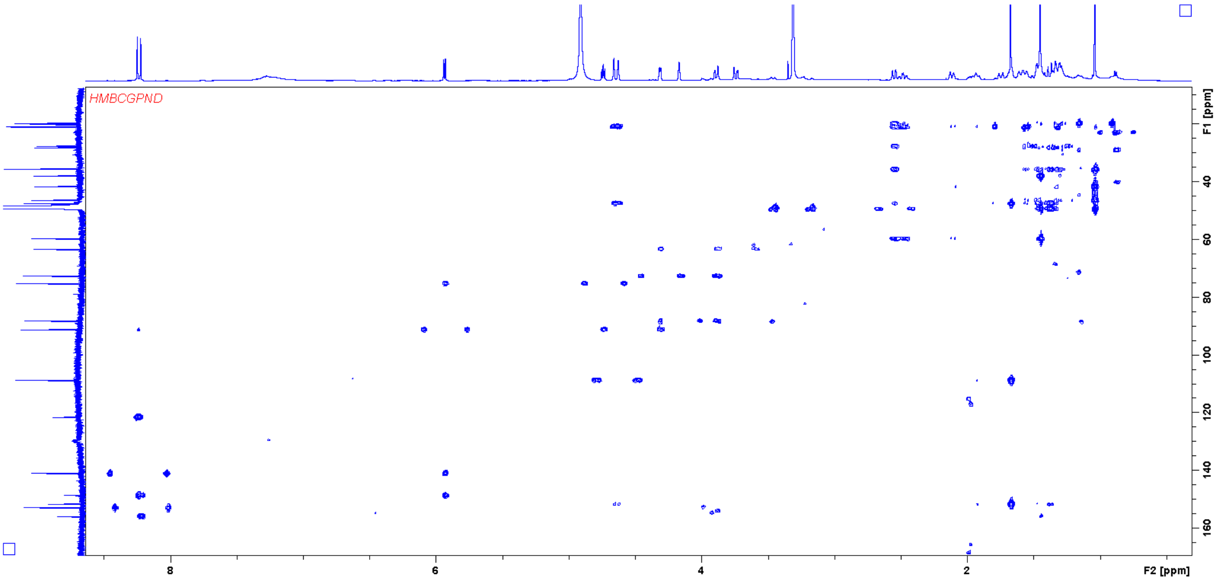

Figure S24. *HMBC* spectrum of sorangiadenosine (**1**) in methanol-d$_4$.

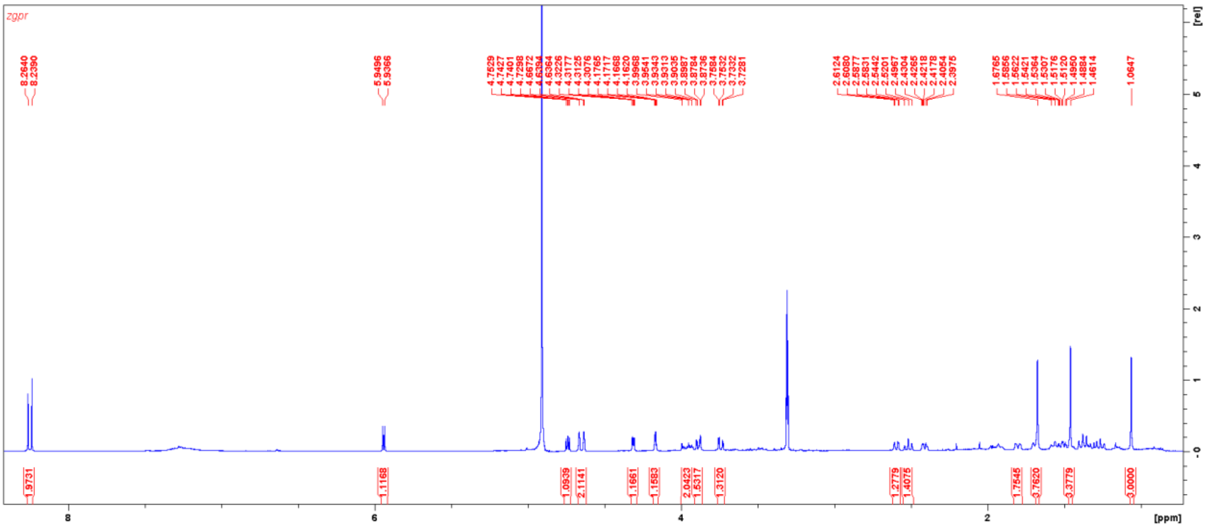

Figure S25. ^{1}*H NMR* spectrum of 2-hydroxysorangiadenosine (**2**) in methanol-d$_4$.

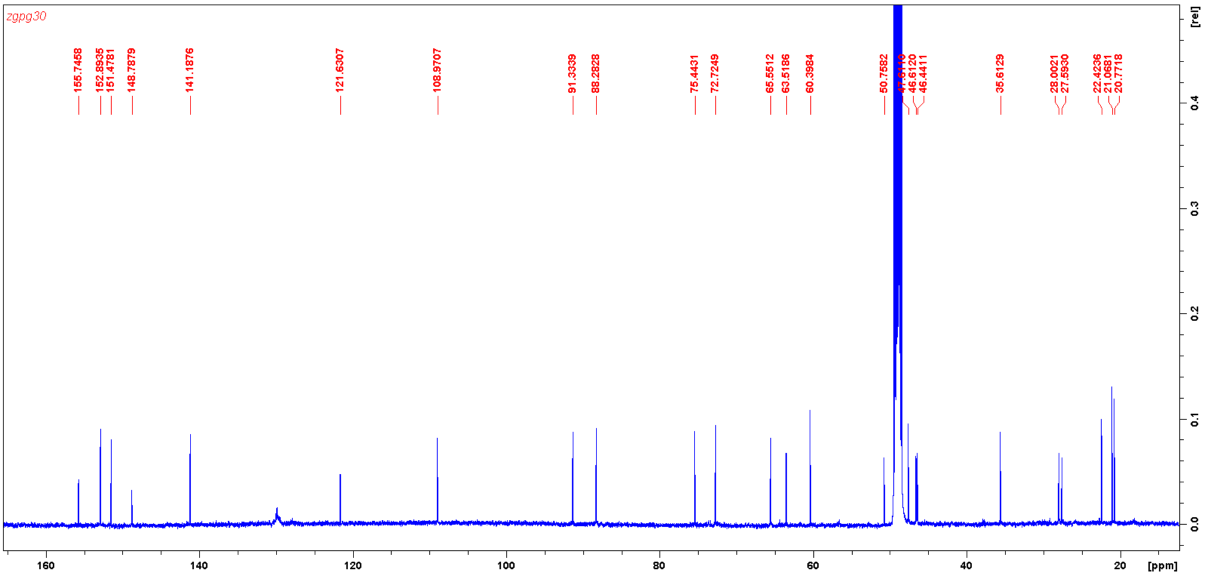

Figure S26. ^{13}*C NMR* spectrum of 2-hydroxysorangiadenosine (**2**) in methanol-d$_4$.

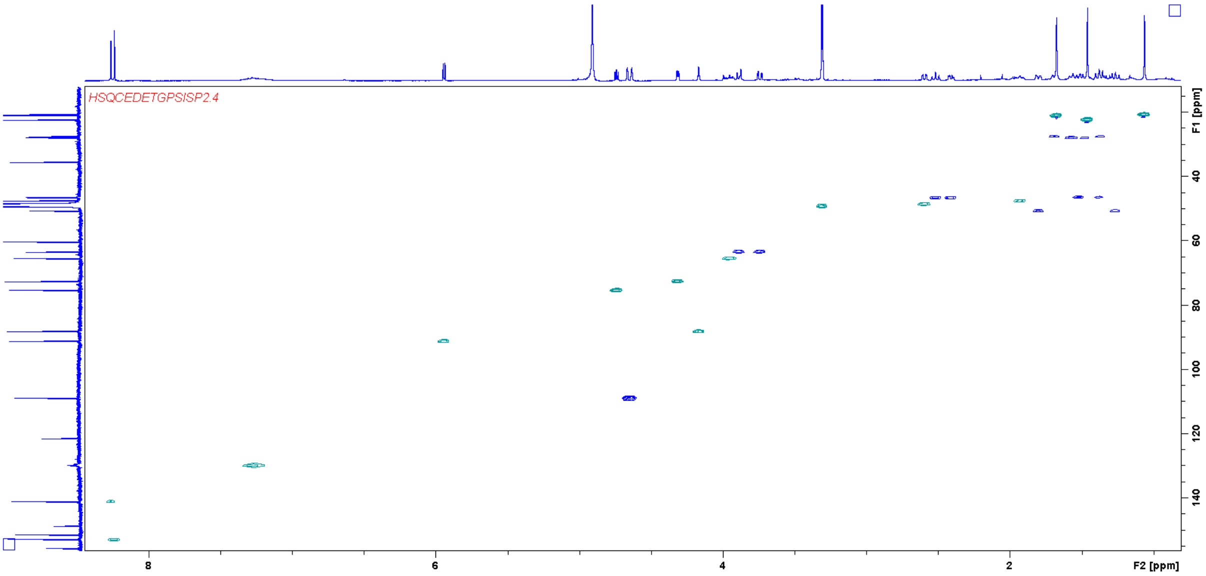

Figure S27. *HSQC* spectrum of 2-hydroxysorangiadenosine (**2**) in methanol-d$_4$.

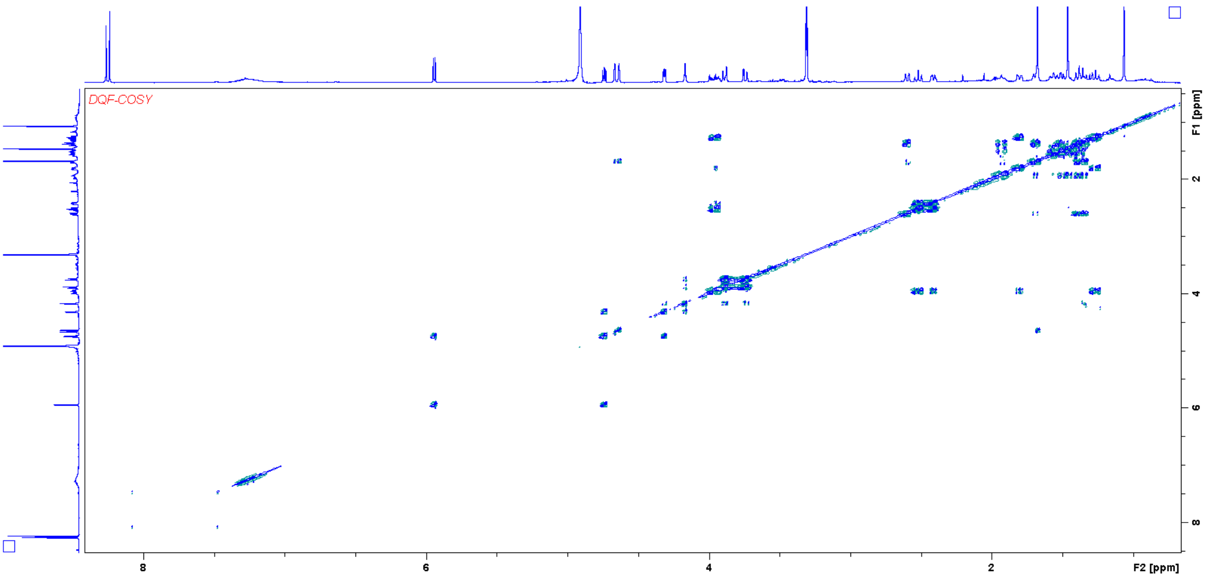

Figure S28. *DQF-COSY* spectrum of 2-hydroxysorangiadenosine (**2**) in methanol-d4.

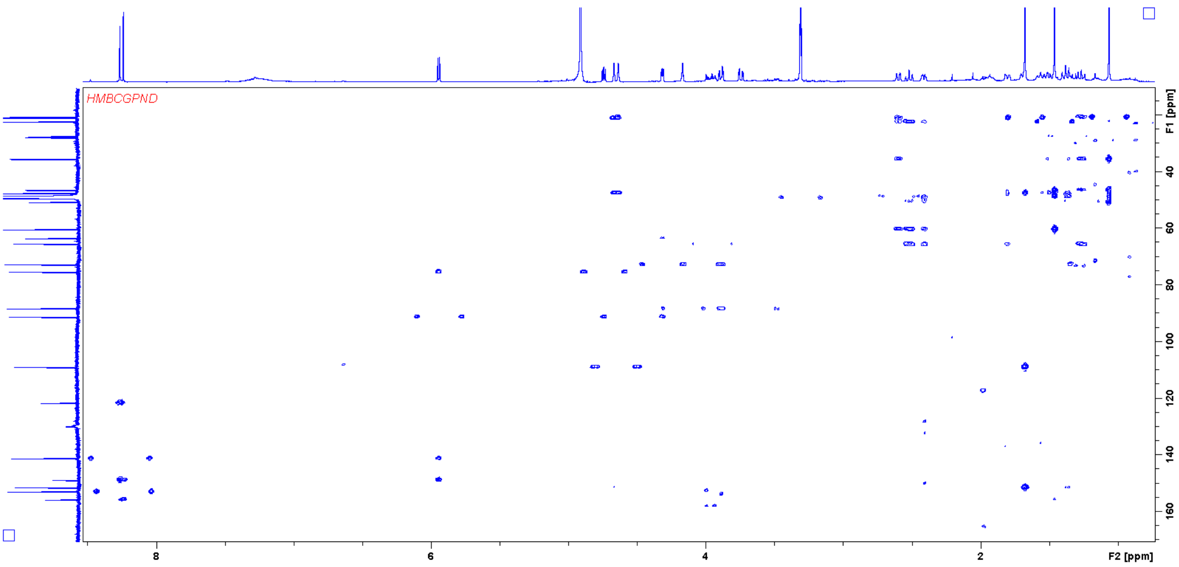

Figure S29. *HMBC* spectrum of 2-hydroxysorangiadenosine (**2**) in methanol-d4.

molecules MDPI

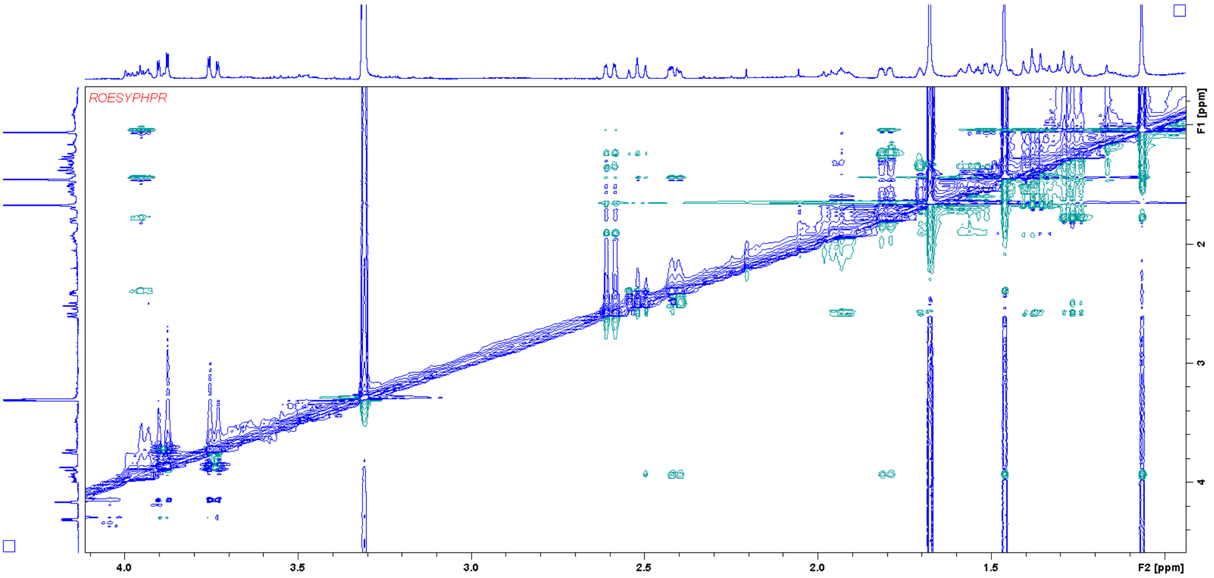

Figure S30. *ROESYphpr* spectrum No. 1 of 2-hydroxysorangiadenosine (**2**) in methanol-d_4.

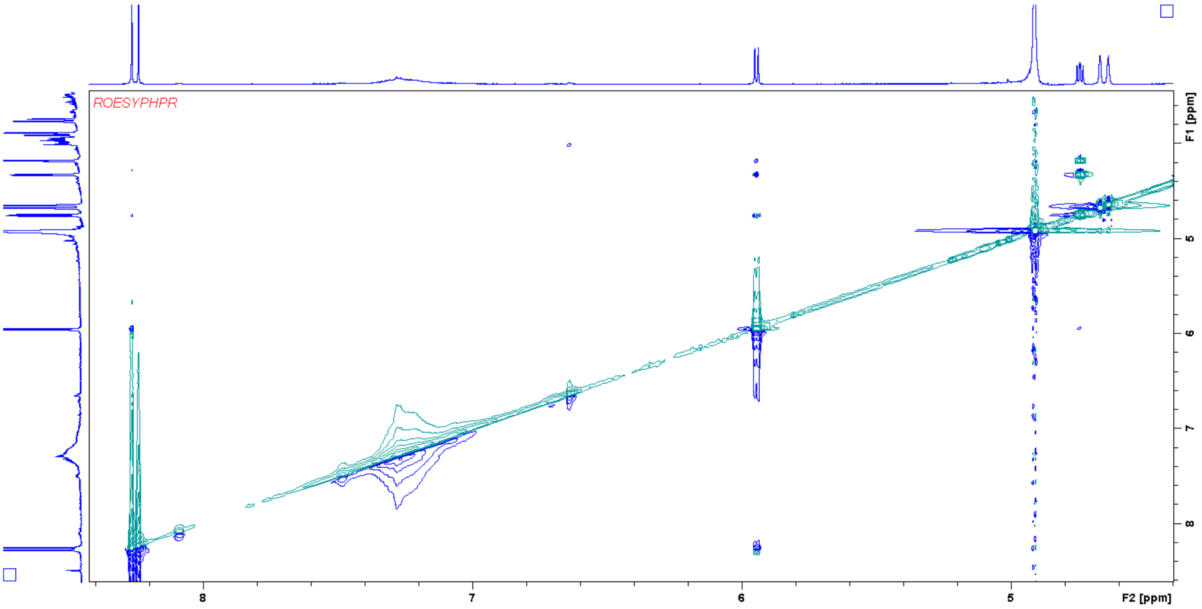

Figure S31. *ROESYphpr* spectrum No. 2 of 2-hydroxysorangiadenosine (**2**) in methanol-d_4.

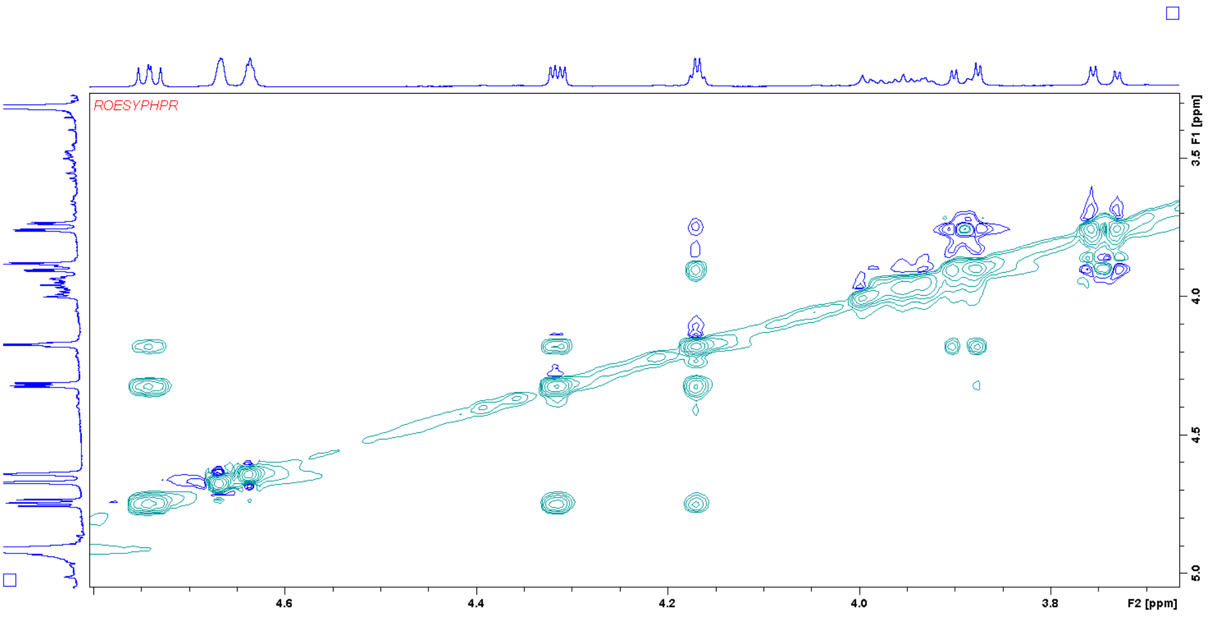

Figure S32. *ROESYphpr* spectrum 3 of 2-hydroxysorangiadenosine (**2**) in methanol-d$_4$.

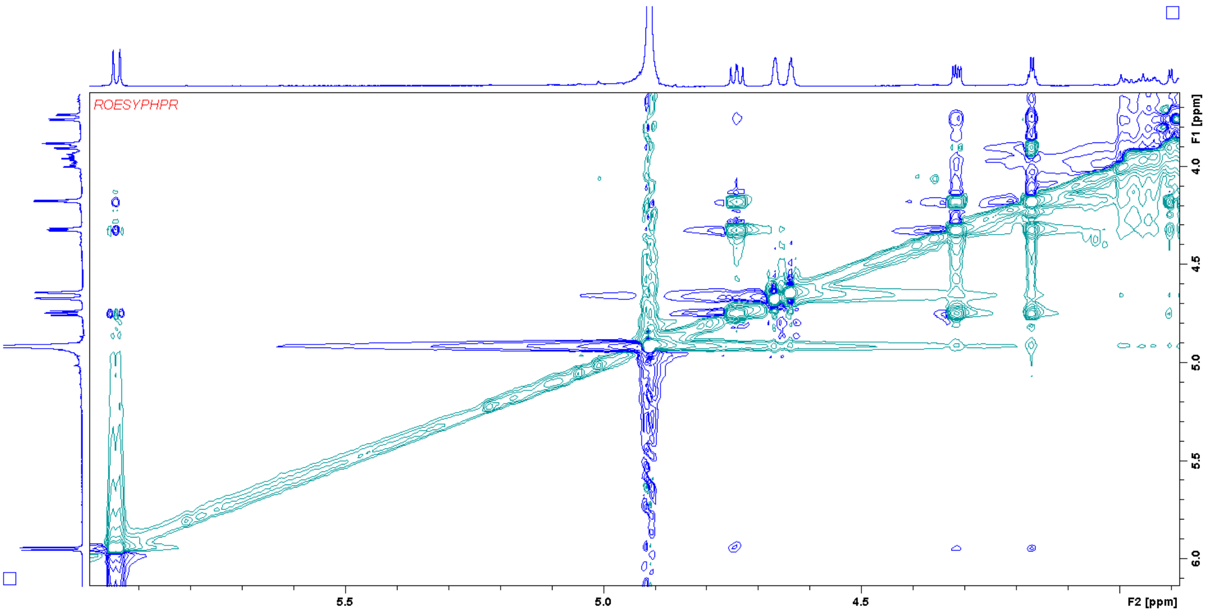

Figure S33. *ROESYphpr* spectrum 4 (expanded region) of 2-hydroxysorangiadenosine (**2**) in methanol-d$_4$.

4. References

1. Bode, H.B.; Ring, M.W.; Schwär, G.; Altmeyer, M.O.; Kegler, C.; Jose, I.R.; Singer, M.; Müller, R. Identification of additional players in the alternative biosynthesis pathway to isovaleryl-CoA in the myxobacterium *Myxococcus xanthus*. *ChemBioChem* **2009**, *10*, 128–140.

2. Li, Y.; Luxenburger, E.; Müller, R. An alternative isovaleryl CoA biosynthetic pathway involving a previously unknown 3-methylglutaconyl CoA decarboxylase. *Angew. Chem. Int. Ed. Engl.* **2012**, *52*, 1304–1308.

3. Mahmud, T.; Wenzel, S.C.; Wan, E.; Wen, K.W.W.; Bode, H.B.; Gaitatzis, N.; Müller, R. A novel biosynthetic pathway to isovaleryl-CoA in myxobacteria: The involvement of the mevalonate pathway. *ChemBioChem* **2005**, *6*, 322–330.

4. Bock, T.; Luxenburger, E.; Hoffmann, J.; Schutza, V.; Feiler, C.; Müller, R.; Blankenfeldt, W. AibA/AibB induces an intramolecular decarboxylation in isovalerate biosynthesis by *Myxococcus xanthus*. *Angew. Chem. Int. Ed.* **2017**, *56*, 9986–9989.

5. Dickschat, J.S. Bacterial terpene cyclases. *Nat. Prod. Rep.* **2015**, *33*, 87–110.

6. Rabe, P.; Rinkel, J.; Klapschinski, T.A.; Barra, L.; Dickschat, J.S. A method for investigating the stereochemical course of terpene cyclisations. *Org. Biomol. Chem.* **2015**, *14*, 158–164.

7. Schifrin, A.; Khatri, Y.; Kirsch, P.; Thiel, V.; Schulz, S.; Bernhardt, R. A single terpene synthase is responsible for a wide variety of sesquiterpenes in *Sorangium cellulosum* So ce56. *Org. Biomol. Chem.* **2016**, *14*, 3385–3393.
